# KDM4B modulates ERα signaling pathway to participate in vascular smooth muscle cell calcification

**DOI:** 10.1038/s41420-025-02765-6

**Published:** 2025-10-07

**Authors:** Fei Liu, Yang Lv, Yanxia Lin, Chunyu Wang, Shengli Wang, Kai Zeng, Baosheng Zhou, Lin Lin, Jianwei Feng, Ge Sun, Xiaocen Chang, Mengsu Cao, Hao Li, Xihong Hu, Shigeaki Kato, Yue Zhao, Wen Tian

**Affiliations:** 1 Department of Geriatrics, the First Hospital of China Medical University., Shenyang City, Liaoning Province China; 2 Department of Geriatrics, Dalian Friendship Hospital Affiliated to Dalian Medical University, Dalian City, Liaoning Province China; 3 Department of Cell Biology, Key Laboratory of Medical Cell Biology, Ministry of Education, School of Life Sciences, China Medical University, Shenyang City, Liaoning Province China; 4 Graduate School of Life Science and Engineering, Iryo Sosei University, Iwaki, Fukushima Japan; 5 Research Institute of Innovative Medicine, Tokiwa Foundation, Iwaki, Fukushima Japan

**Keywords:** Cell biology, Epigenetics, Chromosomes

## Abstract

Vascular calcification (VC) is recognized as an independent predictor of cardiovascular events. Although estrogen replacement is a controversial treatment due to its potential carcinogenic effects, it was considered a protective treatment against VC in postmenopausal women. Estrogen receptor α (ERα) co-regulators were considered as potential therapeutic targets for ERα-related cancers. However, ERα activity and the biological function modulation of ERα co-regulators in VC remain elusive. Histone lysine demethylase 4B (KDM4B) was identified to be highly expressed in human and mouse aortic smooth muscle (ASMC) cells treated with β-phosphoglycerol and in mice overloaded with VitD3 during calcification, as evidenced by western blotting and immunofluorescence staining. Co-immunoprecipitation (Co-IP) was performed to show the association between KDM4B and ERα. Our data demonstrated that KDM4B down-regulated ERα-induced transactivation and that KDM4B depletion increased mRNA expression of endogenous ERα target genes. Furthermore, we provided the evidence to show that KDM4B is associated with Polycomb repressive complex 2 (PRC2) and ERα. In addition, KDM4B depletion decreased the recruitment of PRC2 complex to estrogen response element (ERE) regions of ERα target gene, thereby down-regulating the H3K27me3 levels. Finally, KDM4B-mediated enhancement of ASMCs' calcification was partially attenuated by the estrogen treatment. KDM4B inhibits ERα-induced transactivation independent of its Jumanji-C enzyme active region. Taken together, our study suggests that KDM4B acting as ERα co-repressor is involved in the regulation of VC, indicating that KDM4B may be a new potential therapeutic target for VC treatment.

## Introduction

Vascular calcification (VC) is a common degenerative phenomenon associated with aging, which is common in the end-stage of most chronic diseases and is characterized by the abnormal deposition of calcium and phosphorus onto the vascular wall [1–3]. VC has been recognized as an independent predictor of cardiovascular events [4]. The relationship between vascular calcification and osteoporosis is well-established [5, 6]. Osteoporosis, characterized by excessive bone resorption over formation, leads to the release of calcium into the bloodstream, which can then ectopically deposit in blood vessels, thus promoting vascular calcification. Estrogen is recognized for its role in the prevention of osteoporosis [7] and the inhibition of vascular calcification [8]. Nevertheless, there are clear sex-specific patterns of VC, being present in more than 90% of men and 67% of women over 70 years of age. Coronary artery calcification is a component of VC, and its calcification evaluation is an independent predictor of cardiovascular adverse events in postmenopausal women. In addition to the differential lifestyle habits associated with the sexes, the cardiovascular benefits of women diminish after menopause, with the risk of cardiovascular disease increasing significantly in older women (approximately four times as much as before menopause) [9]. The incidence of coronary artery calcification is lower in premenopausal women than in men, which may be related to the protective effect of estrogen [10]. Estrogen is a vital drug component in preventing and treating postmenopausal osteoporosis, which induces bone formation by activating the estrogen receptor signaling pathway. Mechanically, estrogen has been reported to inhibit the VC process in several ways, including classically promoting E2-related gene transcription, promoting autophagy, and inhibiting the HIF-1α signaling pathway [8, 11, 12]. Although the biological function of the estrogen signaling pathway in VC has been investigated in recent years, the specific molecular mechanism remains unclear.

Estrogen receptor α (ERα) belongs to a nuclear receptor superfamily that undergoes conformational changes and translocates from the cytoplasmic lysate to the nucleus in the presence of estrogen, thereby binding to specific estrogen response element (ERE) to regulate its downstream gene transcription [13]. Studies have shown that ERα/E2 exerts a protective effect against VC by promoting the transcriptional activity of the growth arrest-specific gene 6 (GAS6) [14]. Similarly, receptor activator of nuclear factor-Kappa B ligand (RANKL) promotes VC by regulating the expression of bone morphogenetic protein-2 (BMP2), MGP, and bone-associated protein, which is counteracted by estrogen in a receptor-dependent manner. In addition, estrogen acts mainly through the ERα to counteract these effects of RANKL stimulation [8]. Upon estrogen treatment, activated ERα recruits co-regulators that participate in ERα-mediated transcriptional activation, thereby altering chromatin structure, post-transcriptional modifications, and modulating ERα protein stability [15**–**17].

A variety of ERα co-regulators have been demonstrated to be essential for the development of breast cancer (BC) [18, 19]. The histone-modifying enzymes and chromatin remodeling factors enhance or inhibit ERα-mediated gene transcription, referred to as ERα co-activators or co-repressors. For example, KDM3A, a histone demethylase, plays a crucial role in ERα signaling by regulating the transcription of receptor-target genes through the control of demethylation at the cis-regulatory element H3K9me1/me2. Furthermore, KDM3A has been shown to be necessary for the growth of ERα-positive BC cells [20]. In ERα-positive or ERα-negative BC cells, the deletion of KDM4A reduces the expression of ERα target genes c-Jun and cyclin D1, leading to abnormal cell proliferation [21]. The expression of KDM5A is also up-regulated in BC [22**–**26]. Mechanistically, the accumulation of p16 and p27 is promoted by blocking KDM5A-mediated H3K4me3 demethylation, leading to cell cycle arrest and aging [25]. JMJD6 regulated transactivation of ERα-binding enhancers and their downstream target genes. Furthermore, it is a critical factor in the growth and tumorigenesis of ERα-positive BC cells [27]. These findings indicate that ERα co-regulators are essential for the development of ERα-related diseases, including BC, endometrial carcinomas, and VC. However, the modulation of ERα-induced transactivation and the biological function of ERα co-regulators in VC remain elusive.

JMJD2 family members take active roles in multiple physiological processes, including cell proliferation, migration [28], gene transcription [29], and genome stability [30]. The KDM4 family, a class of demethylases, precisely removes methylation at H3K9 or H3K36 sites, playing a pivotal role in the histone code [28, 31–33]. Lysine demethylase is a class of proteins that removes lysine/arginine methylation at specific histone H3/H4 [34]. Histone lysine demethylase 4B (KDM4B), consisting of 1096 amino acids, exhibits catalytic activity on histone residues H3K9me3, H3K9me2, and H3K36me3, with a preference for the H3K9Me2/3 substrate [32, 35, 36]. KDM4B contains a Jumanji N (JmjN) structural domain, a JmjC structural domain, two plant homology domains, and two Tudor structural domains, among which the active region for demethylase is the JmjC domain [34, 35, 37, 38]. The demethylase enzyme KDM4B, a component of the demethylation module, is involved in gene transcriptional regulation. We have previously demonstrated that KDM4B is recruited to ERα target genes, where it demethylates H3K9me3 and upregulates ERα transcription. Moreover, KDM4B plays an essential role in regulating the estrogen signaling cascade, and its absence inhibits the growth of BC both in vitro and in vivo [39, 40]. Notably, KDM4B itself is also an ERα response gene [41]. Thus, these findings suggest a positive feedback mechanism between KDM4B and ERα, where estrogen-induced KDM4B expression in turn co-regulates and upregulates ERα target genes, thereby promoting BC growth. In addition, KDM4B can induce osteogenic differentiation of mesenchymal stem cells (MSCs). TGF-β induces the expression of KDM4B, whereas KDM4B recruits the transcription factor SMAD3 to the SOX9 promoter to inhibit the H3K9me3 modification level and promote chondrogenesis as well as differentiation of MSCs [42]. In vascular smooth muscle cells (VSMCs), KDM4B can be recruited to the STAT3 binding site of the Runx2 promoter region and inhibit the level of H3K9me3 modification, ultimately promoting Runx2 transcription [43]. Nevertheless, the mechanism underlying the effect of KDM4B on ERα-mediated gene transcription during VC remains poorly understood.

In this study, we explored the potential mechanisms by which KDM4B influences the VC process in vivo and in vitro. KDM4B was highly expressed in the β-phosphoglycerol-induced VC model and a VitD3-induced VC mouse model. Furthermore, KDM4B interacted with endogenous ERα and co-repressed ERα-mediated transactivation. Unexpectedly, luciferase assay results suggest that KDM4B inhibits ERα-mediated gene transcription independent of its JmjC enzyme active region. In addition, KDM4B was required for the recruitment of PRC2 subunits to the ERE on ERα target genes, which was accompanied by the enrichment of H3K27me3 modification levels. Furthermore, KDM4B-mediated enhancement of β-phosphoglycerol (β-GP) induced calcification was attenuated by an estrogen treatment. Overall, our study identified KDM4B as a new ERα cofactor that down-regulates ERα-mediated transactivation in its demethylase-independent manner in human aortic smooth muscle cells (HASMCs) and mouse aortic vascular smooth (MOVAS) cells. Our findings demonstrated that KDM4B promotes VC via the E2/ERα signaling pathway, providing a potential therapeutic strategy for VC in females.

## Results

### KDM4B is highly expressed in β-GP-induced HASMCs and MOVAS cells calcification models

**Fig. 1:**
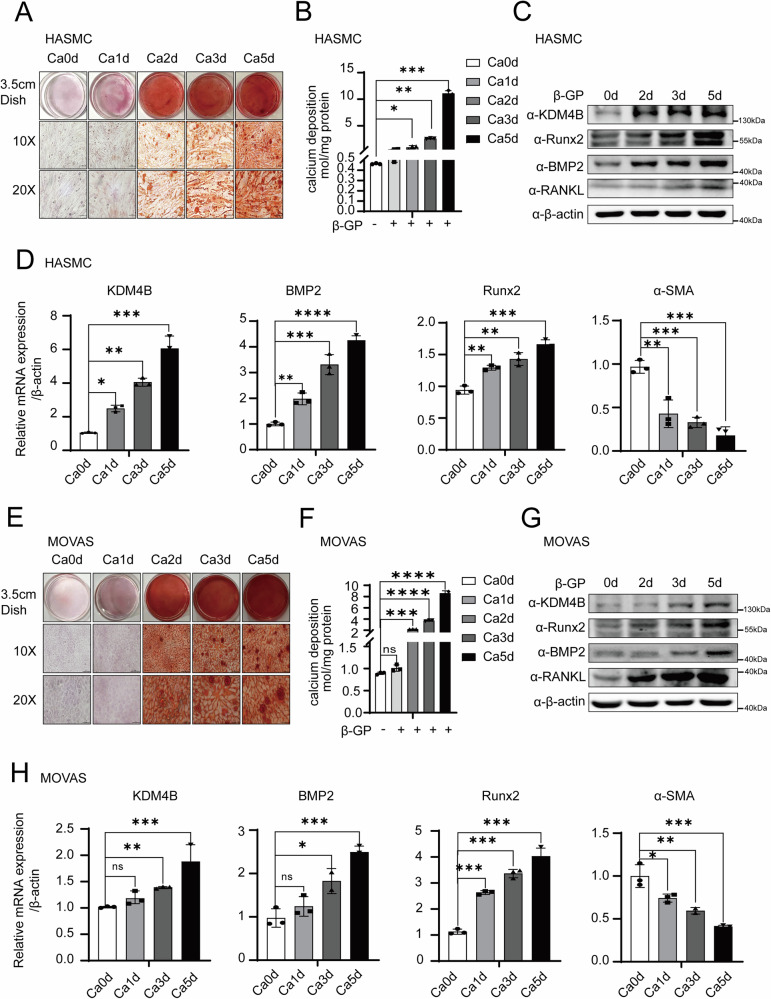
KDM4B is highly expressed in β-GP-induced calcification models in HASMCs and MOVAS cells. **A** Alizarin red staining on different days in Human aortic vascular smooth muscle-derived cells (HASMCs) following 5 mM β-GP treatment. **B** Quantification of calcium deposition on different days in HASMCs treated with 5 mM β-GP. **C** Western blotting analysis for KDM4B, Runx2, BMP, and RANKL expression in HASMCs treated with 5 mM β-GP (β-actin was used as a loading control, and day 0 was set as the control in each parameter). **D** qRT-PCR for KDM4B, Runx2, BMP, and α-SMA expression in HASMCs treated with 5 mM β-GP. Statistical significance was determined using the Student *t*-tests. Error bars represent mean ± SD. *p < 0.05, **p < 0.01, and ns stands for non-significant. **E** Alizarin red staining on different days in MOVAS cells following 5 mM β-GP treatment. **F** Quantification of calcium deposition in MOVAS cells treated with 5 mM β-GP. **G** Western blotting analysis for KDM4B, Runx2, BMP, and RANKL expression in MOVAS cells treated with 5 mM β-GP (β-actin was used as a loading control, and day 0 was set as the control in each parameter). **H** qRT-PCR for KDM4B, Runx2, BMP, and α-SMA expression in MOVAS cells treated with 5 mM β-GP. Statistical significance was determined using the Student *t*-tests. Error bars represent mean ± SD. *p < 0.05, **p < 0.01, and ns stands for non-significant. Data were presented as the mean ± SD of triplicate experiments.

Epigenetic factors that play an important role in the pathogenic regulation of cancer and other diseases’ gene transcription were selected. The expression levels of KDM family genes (KDM2A, KDM3A, KDM4A, KDM5A, KDM6A, and KDM4B) are involved in the epigenetic regulation within HASMCs with different calcification status induced following treatment with 5 mM β-GP for 0, 1, 2, 3, and 5 days. We showed that β-GP induces HASMCs calcification in a time-dependent manner. Simultaneously, the notable expression of KDM4B increased progressively as the duration of calcification extended (as shown in Supplementary Fig. S1A). Alizarin red staining and calcium deposition quantification were performed to measure calcification levels. We further corroborated the finding that β-GP-induced HASMCs calcification in a time-dependent manner (Fig. 1A, B). The expression of calcification-related proteins was measured, with the results suggesting that runt-associated transcription factor 2 (Runx2), BMP2, and RANKL were gradually increasing. We also observed that the expression of KDM4B increased gradually with calcification stimulation time (Fig. 1C). The mRNA levels of calcification-related factors were also analyzed, and the results indicate that Runx2 and BMP2 gradually increased, whereas alpha-smooth muscle actin (α-SMA) gradually decreased. Concurrently, an incremental increase in the mRNA expression of KDM4B was observed across various levels of calcification (Fig. 1D). The same results were obtained for MOVAS cells (Fig. 1E–H). These results suggest that KDM4B is highly expressed in β-GP-induced calcification models and may be involved in the calcification process.

### KDM4B is associated with ERα in HASMCs and MOVAS cells

**Fig. 2:**
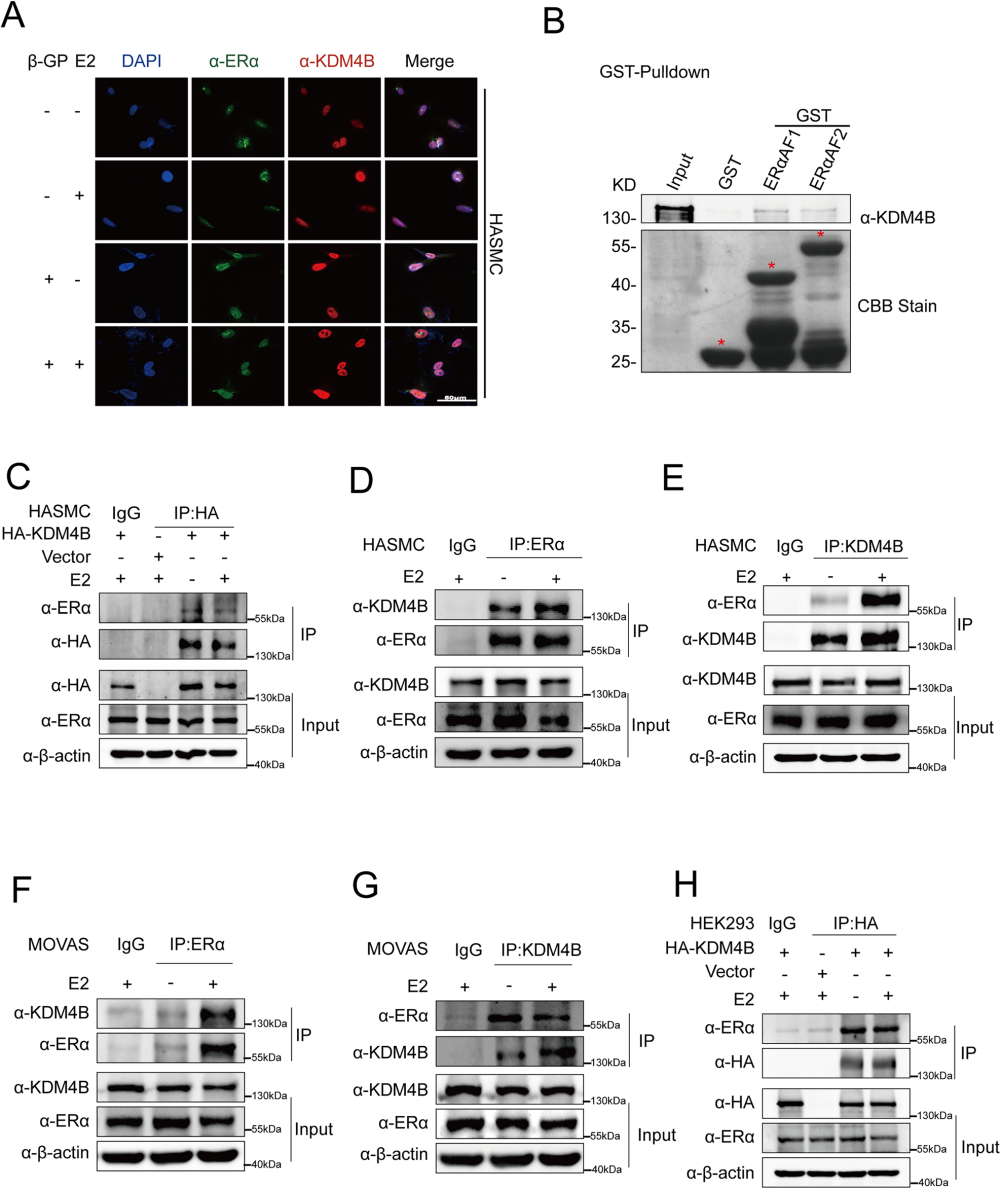
KDM4B associates with ERα in HASMCs and MOVAS cells. **A** Confocal images showing the distribution of KDM4B and ERα in HASMCs after 48-h treatments in the presence of β-GP, either without or with 100 nM E2. This 48-h time point represents an early stage of calcification and was specifically chosen to examine KDM4B’s subcellular localization rather than changes in its expression levels at this stage. Cells were stained for ERα (green) and KDM4B (red), with nuclei counterstained by DAPI (blue) to indicate nuclear morphology. Scale bar = 50 μm. **B** KDM4B directly binds GST-ERαAF1 and GST-ERαAF2 in vitro. The GST-ERαAF1 and GST-ERαAF2 proteins were expressed in prokaryotic cells and purified using GST beads and then incubated with the lysate of HEK-293 T cells expressing HA-KDM4B. After being washed with cold PBS, the eluted complexes were subjected to western blotting and detected with specific antibodies. **C** HASMC cells were co-transfected with PcDNA3.1/HA-KDM4B and ERα plasmids and treated with or without estrogen. Whole-cell extracts were immunoprecipitated with the anti-HA antibody or IgG after transfection for two days. Input means 5% of the entire extract for each column. **D**, **E** Co-IP experiments show endogenous KDM4B and ERα associated with each other in HASMC cells. Reciprocal Co-IP and immunoblotting were performed with antibodies as indicated. A 5% fraction of the input cell lysate before immunoprecipitation was loaded as a control. **F**, **G** Co-IP experiments show the interaction between endogenous KDM4B and ERα in MOVAS cells. **H** HEK-293 cells were co-transfected with PcDNA3.1/HA-KDM4B and ERα plasmids and treated with or without estrogen (E2, 100 nM). Whole-cell extracts were immunoprecipitated with the anti-HA antibody or IgG after transfection for two days. Input means 5% of the whole extract for each column.

Clinical findings showed that women have a higher risk of VC and cardiovascular disease after menopause [9]. Furthermore, the ERα signaling pathway has been suggested to be involved in inhibiting the calcification process associated with VC [8]. KDM4B is also a critical epigenetic demethylase involved in regulating transcription factors, and it upregulates ERα-target genes; hence, sustaining BC cell growth. In our effort to understand the regulatory relationship between KDM4B and ERα in VC, we investigated the association between these two proteins. Initially, immunofluorescence assays were performed to examine the subcellular distribution of KDM4B and ERα in HASMCs. Our data showed that KDM4B and ERα are predominantly distributed in the nucleus in the absence or presence of E2 treatment in HASMCs (Fig. 2A). GST-labeled ERαAF1 or ERαAF2 expression vectors were further used for the GST pull-down experiments, revealing that KDM4B directly binds to GST-ERαAF1 and AF2 in vitro. (Fig. 2B). In addition, HASMCs were transfected with the KDM4B expression plasmid for the co-IP assay. Co-IP results showed that HA-labeled KDM4B precipitated with ERα (Fig. 2C).

Further, endogenous interactions between KDM4B and ERα in HASMCs were detected using endogenous-cellular co-IP assays (Fig. 2D, E). Consistent with the previous results, endogenous interactions between KDM4B and ERα were also observed in MOVAS cells (Fig. 2F, G). HEK-293 cells were co-transfected with ERα and HA-labeled KDM4B expression plasmid for the co-IP assay. Our results showed that ERα precipitated with the HA-labeled KDM4B (Fig. 2H). These data demonstrated that KDM4B associates with ERα in HASMCs and MOVAS cells.

### KDM4B co-represses ERα-induced transactivation in HASMCs and MOVAS cells

**Fig. 3:**
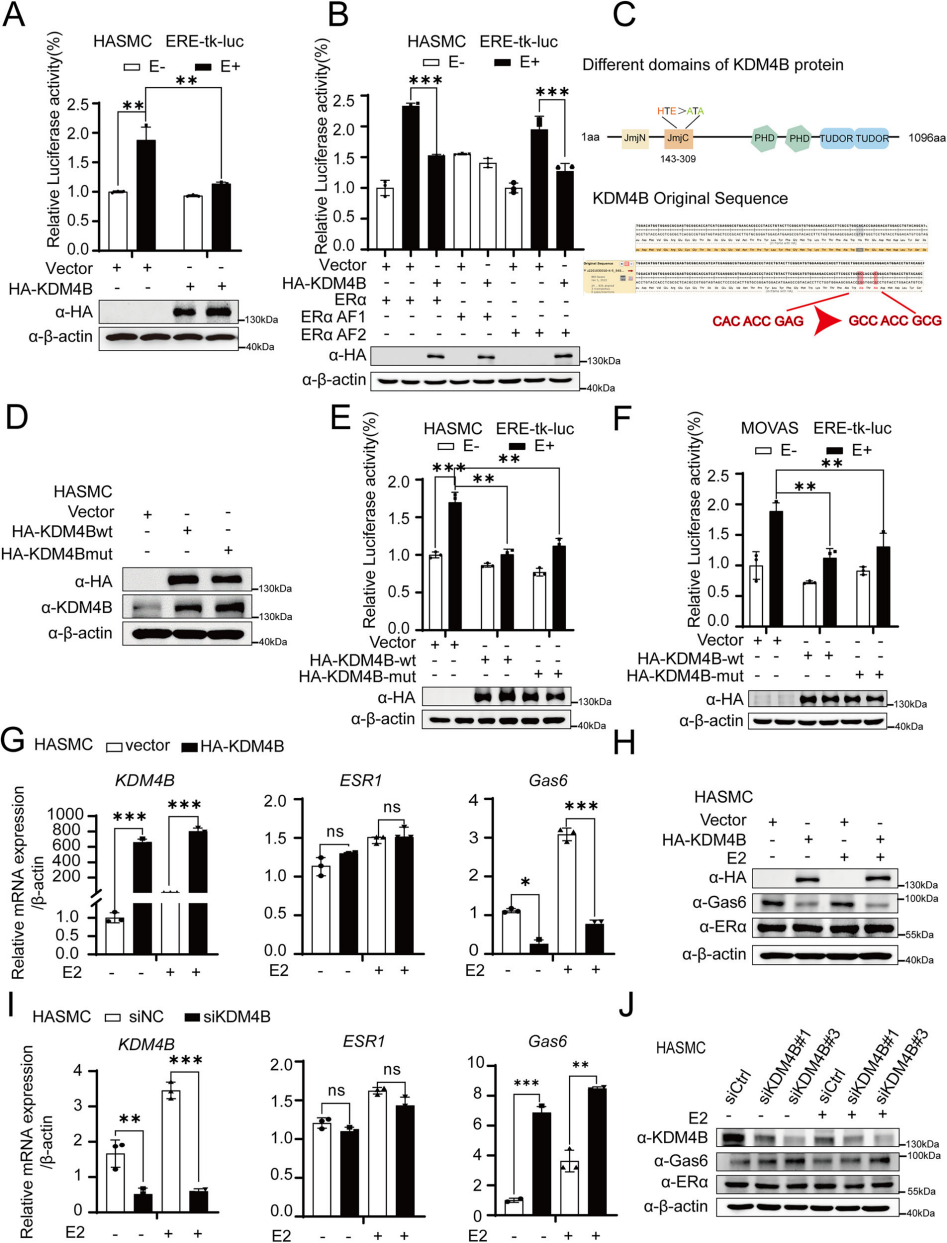
KDM4B co-represses ERα-induced transactivation independent of its demethylase activity in HASMCs and MOVAS cells. **A** KDM4B co-represses ERα-induced transactivation. HASMCs were co-transfected with ERE-tk-luc and pRL-tk expression plasmids together with HA-KDM4B or PcDNA3.1 plasmid (vector) as indicated and treated with or without E2. The expression levels of HA-KDM4B were detected using western blotting with anti-HA. **B** KDM4B co-represses the transcriptional activity induced by ERα or its two truncated mutants (ERαAF1 and ERαAF2) in HASMCs. **C** Plasmid sequencing diagram of KDM4B and KDM4Bmut (HTE/ATA) (the loss-of-function mutation in KDM4B’s demethylase active site). **D** HASMCs were transfected with HA-KDM4Bwt (overexpressing KDM4B full-length) and the HA-KDM4B mutant (HTE/ATA) and cultured in growth medium for three days. **E** KDM4B (HTE/ATA), carrying a loss of function in demethylase activity, exhibits reduced coactivation function on ERα action in HASMCs. **F** KDM4B (HTE/ATA), carrying a loss of function in demethylase activity, exhibits reduced coactivation function on ERα action in MOVAS cells. Relative luciferase activity represents the mean value at least three replicates. Student *t*-tests were used, and error bars represent mean ± SD. *p < 0.05, **p < 0.01, and ns stands for non-significant. **G**, **I** qPCR analysis was examined for the effects of KDM4B on mRNA (Gas6) expression of endogenous ERα target genes in HASMCs. Statistical significance was determined using the Student *t*-tests. Error bars represent mean ± SD. *p < 0.05, **p < 0.01, and ns stands for non-significance. **H**, **J** The effects of KDM4B on protein (Gas6) expression of endogenous ERα target genes in HASMCs. β-actin was used as a control.

To detect the regulatory function of KDM4B on the ERα activity, luciferase assays were performed in HASMCs. Our results showed that KDM4B significantly down-regulates ERα-mediated transactivation (Fig. 3A). To further identify the ERα domain regulated by KDM4B, we performed a luciferase experiment that examined the effect of KDM4B on ERα-induced transcriptional activity containing a nonligand-dependent AF1 domain (ERα AF1) or a ligand-dependent AF2 domain (ERα AF2). We observed that KDM4B down-regulated ERα AF2-mediated transcriptional activation in the presence of E2, whereas the ERα AF1 activation was not regulated (Fig. 3B). To further detect whether the demethylase activity of KDM4B is required for its regulation function on ERα-mediated transactivation, according to the previous paper [44], we constructed a KDM4B mutant expression plasmid (KDM4B HTE/ATA) carrying a demethylase loss-of-function mutation, as shown in diagram of plasmid sequencing (Fig. 3C). Western blotting was used to verify the transfection efficiency of KDM4B full-length and its mutant counterpart (Fig. 3D). Alternatively, the luciferase assay was performed to detect whether KDM4B down-regulates ERα-mediated transactivation depending on its demethylase activity. We observed that both KDM4B full-length and its mutant significantly down-regulated ERα-mediated transactivation in both HASMCs and MOVAS cells. This suggests that KDM4B may down-regulate ERα-mediated transactivation independent of its demethylase activity (Fig. 3E, F). To strengthen the argument, a luciferase assay was also performed with the KDM4B inhibitor; the results showed that the KDM4B inhibitor has similar effects as that of KDM4B or the KDM4B mutant on ERα-mediated transactivation (Supplementary Fig. S2A). Meanwhile, the results from Alizarin red staining showed that KDM4Bwt, KDM4B mutant, or KDM4B inhibitor aggravated β-GP-induced calcification in MOVAS cells, respectively (Supplementary Fig. S2B). These data suggest that the co-repression function of KDM4B on ERα-mediated transactivation may not be dependent on its demethylase activity. Furthermore, qPCR was used to examine the modulation functions of KDM4B on the expression of the endogenous estrogen-responsive gene mRNA in HASMCs. Our data showed that ectopic expression of KDM4B decreased the mRNA expression of *Gas6*, but not *ESR1,* in HASMCs and MOVAS cells (Fig. 3G and Supplementary Fig. S3A). Furthermore, western blotting showed that KDM4B overexpression decreased Gas6 protein expression in HASMCs and MOVAS cells (Fig. 3H and Supplementary Fig. S3C). Similar experiments were performed in HASMCs and MOVAS cells transfected with siKDM4B; we observed that KDM4B depletion exceedingly increased the mRNA and protein expression of Gas6 (Fig. 3I, J, and Supplementary Fig. S3B, D). Furthermore, both overexpression of the KDM4B full-length and the mutant version decreased Gas6 protein expression in HASMCs (Supplementary Fig. S3E). In summary, our results suggest that KDM4B co-represses ERα-induced transactivation independent of its demethylase activity in HASMCs and MOVAS cells.

### KDM4B facilitates the recruitment of PRC2 core proteins to the *cis*-regulatory elements of ERα target genes

**Fig. 4:**
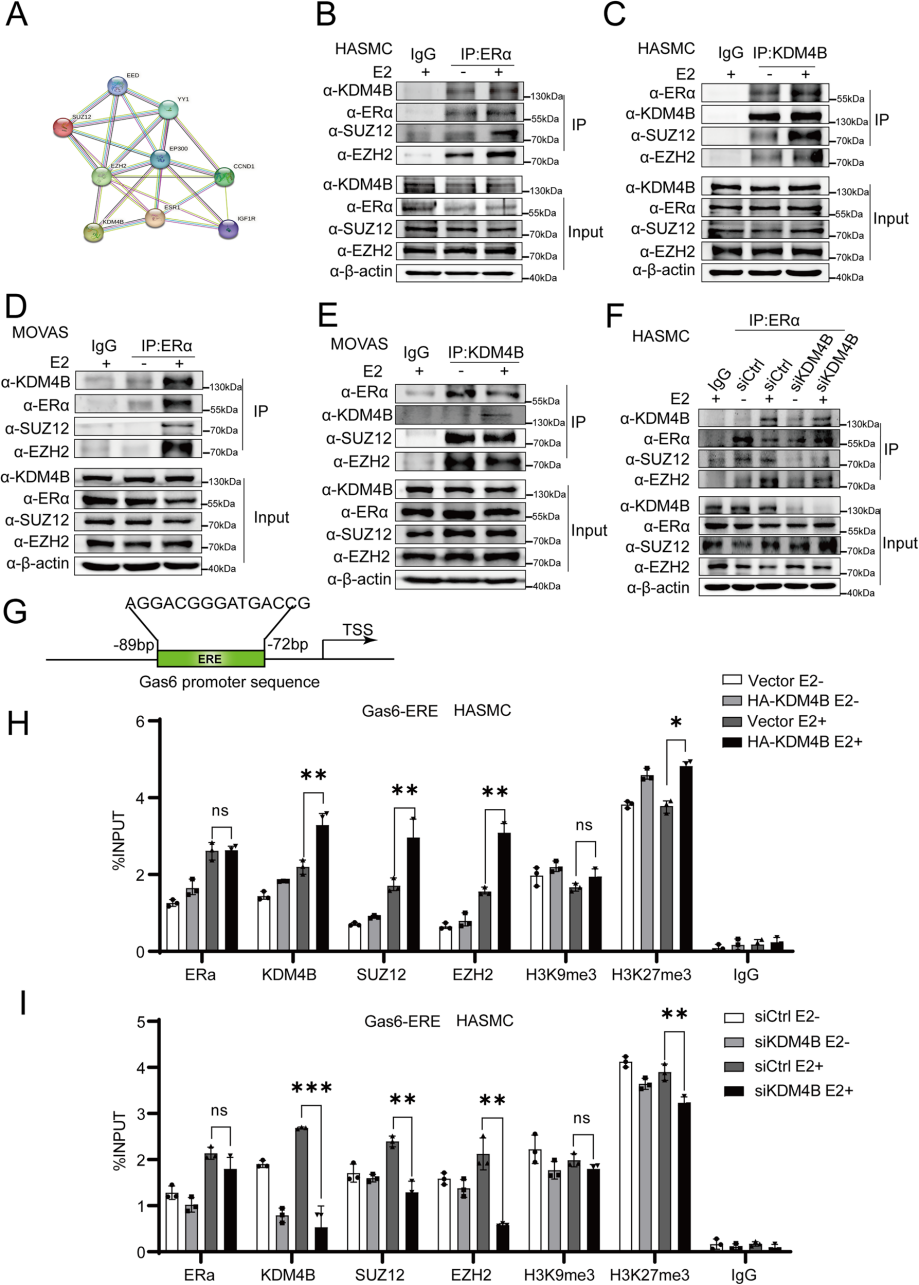
KDM4B facilitates the recruitment of PRC2 core proteins to the *cis*-regulatory elements of ERα target genes. **A** The STRING protein network analysis suggests that KDM4B might associate with ERα and the subunits of the PRC2 complex (PRC2 core proteins), including EZH2 and SUZ12. **B**, **C** The Co-IP assays show an association between ERα and PRC2 core proteins and KDM4B in HASMCs. **D**, **E** The Co-IP assays show an association between ERα and PRC2 core proteins and KDM4B in MOVAS cells. **F** The Co-IP experiment in HASMCs harboring control siRNA or siKDM4B was performed using anti-ERα and IgG. Precipitated proteins were determined by western blotting with antibodies against PRC2 complex core proteins or KDM4B/ERα as indicated. The location of the star indicates where the protein was expressed. **G** A diagram indicating the KDM4B/ERα enrichments and modification of H3K9me3 at an independent ERE upstream of the TSS region of the Gas6 gene. The location of the EREs is indicated in the diagram above (Green rectangle). **H**, **I** ChIP assays were conducted to show the effect of KDM4B on the recruitment of PRC2 (EZH2/SUZ12) to the Gas6 promoter ERE and the associated changes in H3K27me3 or H3K9me3 levels. ChIP assays for HASMCs transfected with an overexpression construct for KDM4B, and either control siRNA or siKDM4B, following treatment with or without estrogen (100 nM).

According to a previous report, KDM4B upregulates ERα-mediated gene transcription mainly in BC [40]. Interestingly, our results demonstrate that KDM4B down-regulates ERα-mediated gene transcription. Thus, we would like to further study the mechanism through which KDM4B is involved in ERα-mediated gene transcription in VC. We sought to identify the proteins interacting with KDM4B as potential protein complexes. The STRING protein network analysis suggests that KDM4B may be associated with ERα and the subunits of the PRC2 complex (PRC2 core protein), including Enhancer of Zeste homolog 2 (EZH2) and Suppressor of Zeste 12 (SUZ12) (Fig. 4A). To determine the correlation of ERα and PRC2 core proteins and KDM4B in HASMCs and MOVAS cells, we performed co-IP experiments. Our data demonstrated that ERα and KDM4B associated with the core protein subunits of the PRC2 complex (Fig. 4B–E). Additionally, the interactions between ERα and the PRC2 subunits were significantly reduced when KDM4B was depleted (Fig. 4F). These results suggest that KDM4B could mediate the association of ERα and the PRC2 core protein.

To further analyze the molecular mechanism underlying the influence of KDM4B on estrogen-induced gene transcription, specifically for genes such as *Gas6*, we utilized the JASPAR software to predict the Estrogen response element (ERE) on *Gas6*. An ERE spanning between −72 and −89 bp was observed from the translation start site of *Gas6* (Fig. 4G). A ChIP assay was performed in HASMCs treated with or without estrogen. During estrogen induction, KDM4B or ERα was recruited into the promoter region of Gas6-ERE. The overexpression of KDM4B promoted the recruitment of PRC2 core protein, not affecting the recruitment of ERα; thus, enhancing the levels of H3K27me3 proximal to the Gas6-ERE region (Fig. 4H). To confirm these results, ChIP analysis was further performed in cells transfected with siKDM4B. KDM4B deletion can reduce the recruitment of the PRC2 complex to the same region. In addition, in the presence of E2, KDM4B deletion decreased the levels of H3K27me3 adjacent to the Gas6-ERE promoter region, whereas no significant effects of KDM4B on H3K9me3 levels were observed (Fig. 4I). These results indicated that KDM4B could facilitate PRC2 subunit recruitment to the promoter region of ERα target genes; thus, altering the level of H3K27me3 and enhancing gene transcription. In summary, our results suggest that KDM4B is associated with ERα and PRC2 core proteins and down-regulates ERα-mediated gene transcription in HASMCs and MOVAS cells.

### KDM4B aggravates β-GP-induced calcification in HASMCs and MOVAS cells

**Fig. 5:**
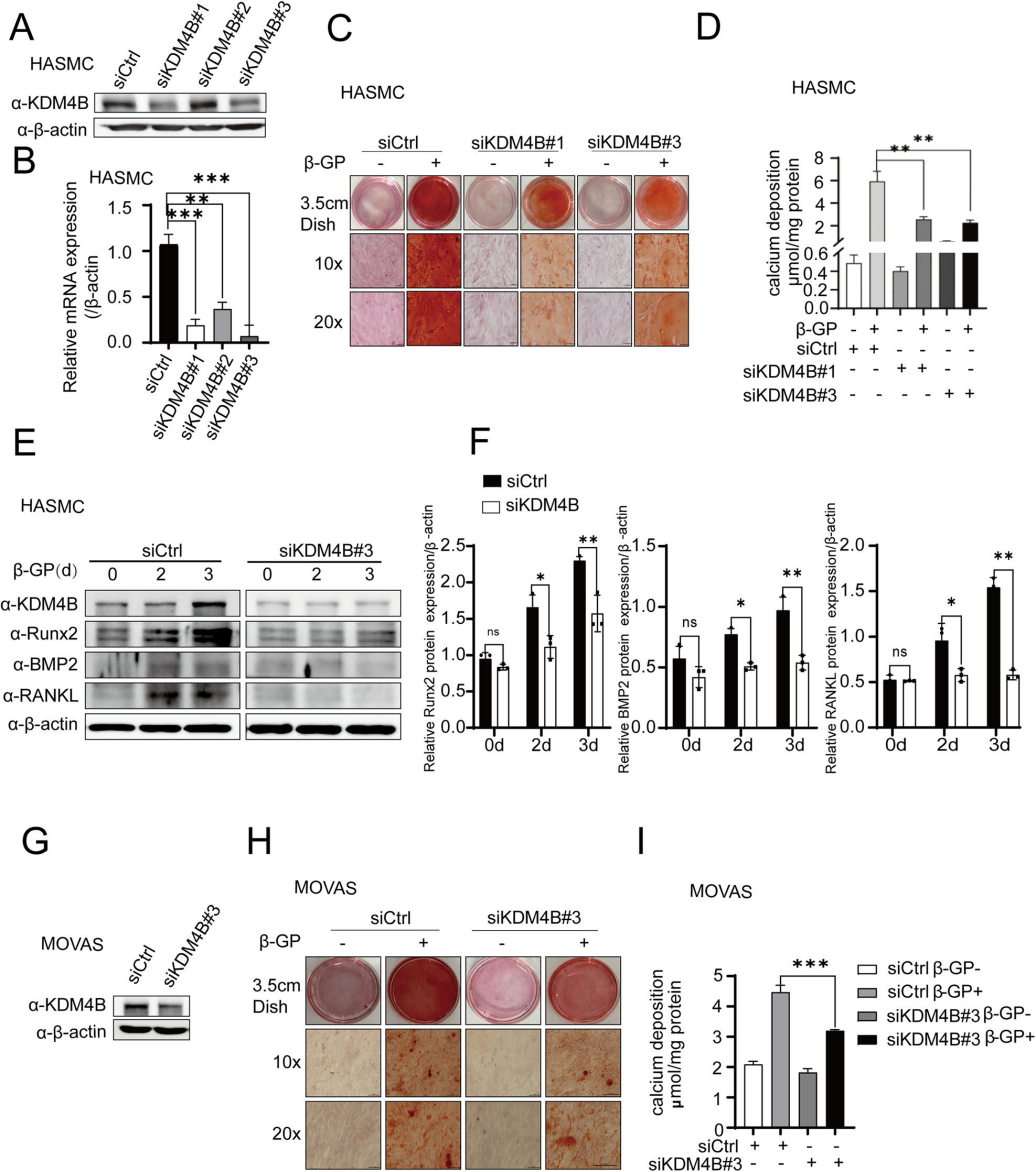
KDM4B depletion alleviates β-GP-induced calcification in HASMCs and MOVAS cells. All groups were treated with or without 5 mM β-GP for five days before quantifying the calcification levels. **A**, **B** The depletion of KDM4B was achieved using three different siRNAs (siKDM4B#1, siKDM4B#2, and siKDM4B#3) and was detected through western blotting and quantitative real-time PCR (qPCR) in HASMCs. **C** Alizarin red staining of HASMCs transfected with siKDM4B#1 and siKDM4B#3. **D** Quantifying calcium deposition in HASMCs transfected with siKDM4B#1 and siKDM4B#3. **E**, **F** Western blotting analysis and quantification of protein expression for KDM4B, Runx2, BMP, and RANKL expression in HASMCs (β-actin was used as a loading control). The KDM4B knockdown was used as the control for each parameter. **G** The depletion of KDM4B in MOVAS cells was achieved using siKDM4B#3 and was detected through western blotting. **H** Alizarin red staining in MOVAS cells transfected with siKDM4B#3. **I** Quantification of calcium deposition in MOVAS cells transfected with siKDM4B#3. Data were collected from 3 replicates and expressed as the mean ± SD. *p < 0.05, **p < 0.01, and ns stands for non-significant.

We showed that KDM4B acts as a co-repressor of ERα and mediates the ERα transactivation in HASMCs and MOVAS cells. Further, we examine the biological function of KDM4B in β-GP-induced calcification in HASMCs and MOVAS cells. KDM4B was knocked down following transfection with siKDM4B. To mitigate potential off-target effects of siRNA, we analyzed the impact of two distinct siRNA sequences (siKDM4B#1 and siKDM4B#3) on KDM4B, as verified by western blotting and qPCR in HASMCs (Fig. 5A, B) and MOVAS cells (Fig. 5G). Concurrently, Alizarin red staining and quantitative calcium analyses revealed that KDM4B depletion alleviated the calcification induced by the 5 mM β-GP treatment in HASMCs and MOVAS cells (Fig. 5C, D, H, I). Aligning with these findings, western blotting analysis and subsequent quantification of protein expression demonstrated that KDM4B depletion led to a decrease in the expression of calcification markers such as Runx2, BMP, and RANKL over different treatment periods with 5 mM β-GP in HASMCs (Fig. 5E, F).

**Fig. 6:**
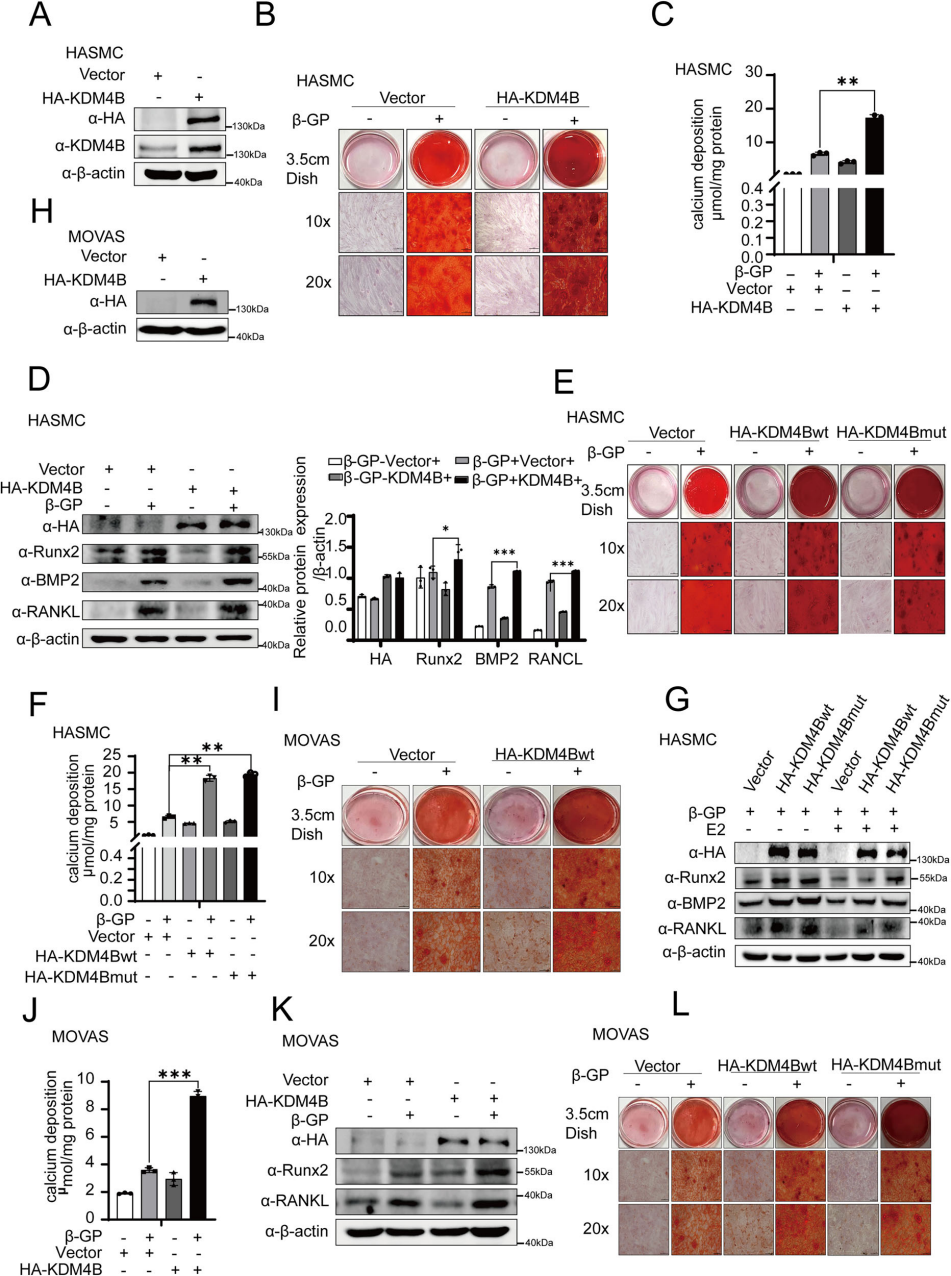
Ectopic expression of KDM4B aggravates β-GP-induced calcification in HASMCs and MOVAS cells. **A** Western blotting of HASMCs overexpressing KDM4B following transfection with the HA-KDM4B. **B**, **E** Alizarin red staining of HASMCs. **C**, **F** Quantification of calcium deposition in HASMCs. **D**, **G** Western blotting analysis for HA-KDM4B, Runx2, BMP2, RANKL, and β-actin (used as a loading control). The KDM4Bwt or KDM4Bmut were overexpressed in HASMCs treated with β-GP and used as the control condition for each parameter. **H** Western blotting of MOVAS cells overexpressing KDM4B following transfection with the HA-KDM4B. **I**, **L** Alizarin red staining of MOVAS cells. **J** Quantification of calcium deposition in MOVAS. **K** Western blotting analysis and quantification of protein expression for HA-KDM4B, Runx2, RANKL, and β-actin (used as a loading control). The KDM4Bwt or KDM4Bmut were overexpressed in MOVAS cells treated with β-GP and used as the control condition for each parameter. Data were collected from 3 replicates and expressed as the mean ± SD. *p < 0.05, **p < 0.01, and ns stands for non-significant.

To further reveal the role of KDM4B in calcification, western blotting analyses were performed in cells with ectopic expression of KDM4B (Fig. 6A, H). Alizarin red staining and calcium deposition indicate that KDM4B overexpression significantly aggravated β-GP-induced calcification in HASMCs (Fig. 6B, C). Furthermore, we observed enhancing calcification effects for both KDM4B full-length and KDM4B mutant overexpression (Fig. 6E, F). Accordingly, the western blot analysis of calcification markers, including Runx2, BMP2, and RANKL, indicates that both KDM4Bwt and KDM4Bmut exert comparable effects on β-GP-induced calcification in HASMCs, as illustrated in Fig. 6D, G. This increase in marker expression was also observed over different days of the 5 mM β-GP treatment in HASMCs, as confirmed by western blotting analysis and subsequent quantification of protein expression (Supplementary Figs. S5A, 4B). These effects were also observed in MOVAS cells (Fig. 6I, J, K, L). Taken together, our results suggest that KDM4B may play an essential role in calcification, and this function may not be influenced by its deubiquitinase activity.

### KDM4B-mediated enhancement of β-GP-induced calcification is partially attenuated by estrogen treatment

**Fig. 7:**
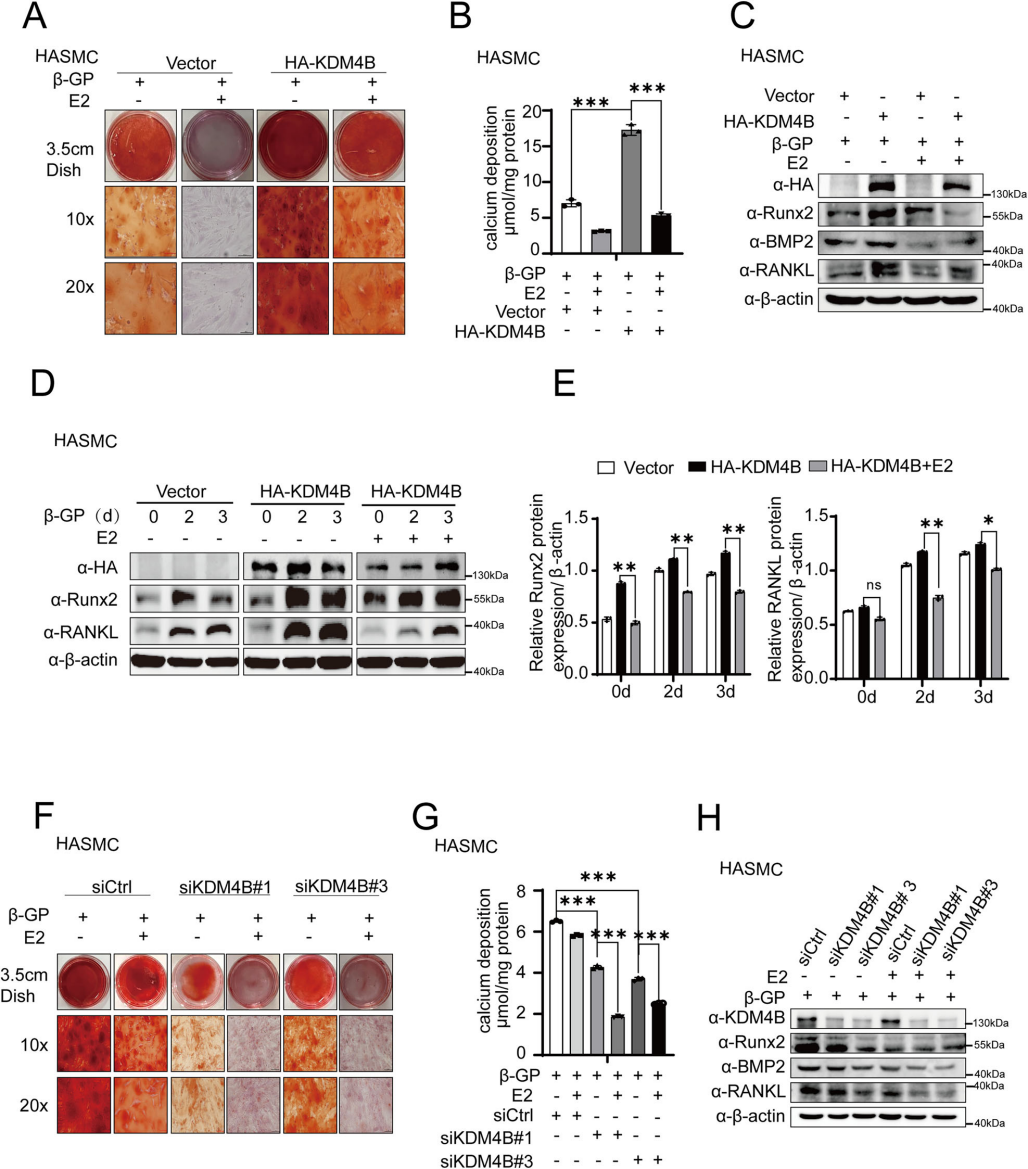
KDM4B-mediated enhancement of β-GP-induced calcification is attenuated by estrogen treatment. **A**, **B** Alizarin red staining and calcium quantification in HASMCs overexpressing KDM4B vs. control, with 5 mM β-GP and ±E2 treatment. Data were collected from 3 replicates and expressed as the mean ± SD. **C**–**E** Western blotting analysis and quantification of protein expression for KDM4B, Runx2, BMP2, RANKL, and β-actin (used as a loading control). HASMCs with KDM4B knockdown followed by treatment with or without β-GP were used as the control for each parameter. **F**, **G** Alizarin red staining and Quantification of calcium deposition of HASMCs show that calcification outcomes in KDM4B-knockdown HASMCs vs. control, under the same +β-GP, ±E2 conditions. **H** Western blotting analysis and quantification of protein expression for KDM4B, Runx2, BMP2, RANKL, and β-actin (β-actin was used as a loading control). HASMCs with KDM4B knockdown followed by β-GP treatment were used as the control for each parameter. Data are expressed as the mean ± SD of triplicate experiments. *p < 0.05, **p < 0.01, and ns stands for non-significant.

The ERα signaling pathway is essential for suppression of VC. Having established in this study that KDM4B acts as a co-repressor of ERα, inhibiting ERα-mediated transactivation. Next, we thus turned to examine whether KDM4B-mediated enhancement of β-GP-induced calcification is related with ERα signaling. Alizarin red staining and quantitative calcium analysis showed that KDM4B overexpression increased calcification following the β-GP treatment, which was partially attenuated by the estrogen treatment (Fig. 7A, B). In addition, the pro-calcific effect of KDM4B overexpression on β-GP-induced calcification, as detected by the expression of calcification markers Runx2, BMP2, and RANKL, was attenuated by estrogen treatment in HASMCs (Fig. 7C, D, E). Additionally, Alizarin red staining and quantitative calcium analysis revealed that KDM4B depletion reduced calcification in the presence of the β-GP treatment; the estrogen treatment at least partially attenuates KDM4B-mediated enhancement of β-GP-induced calcification (Fig. 7F, G). Western blotting results showed that KDM4B depletion could decrease β-GP-induced calcification, indicated by the expression of calcification markers Runx2, BMP2, and RANKL was also attenuated by the estrogen treatment in HASMCs or MOVAS (Fig. 7H, Supplementary Fig. 5A, B). These results suggest that the function of KDM4B on β-GP-induced calcification is, if not all, but partially related to the estrogen/ERα signaling pathway.

### KDM4B is highly expressed in a VitD3-induced calcification mouse model

**Fig. 8:**
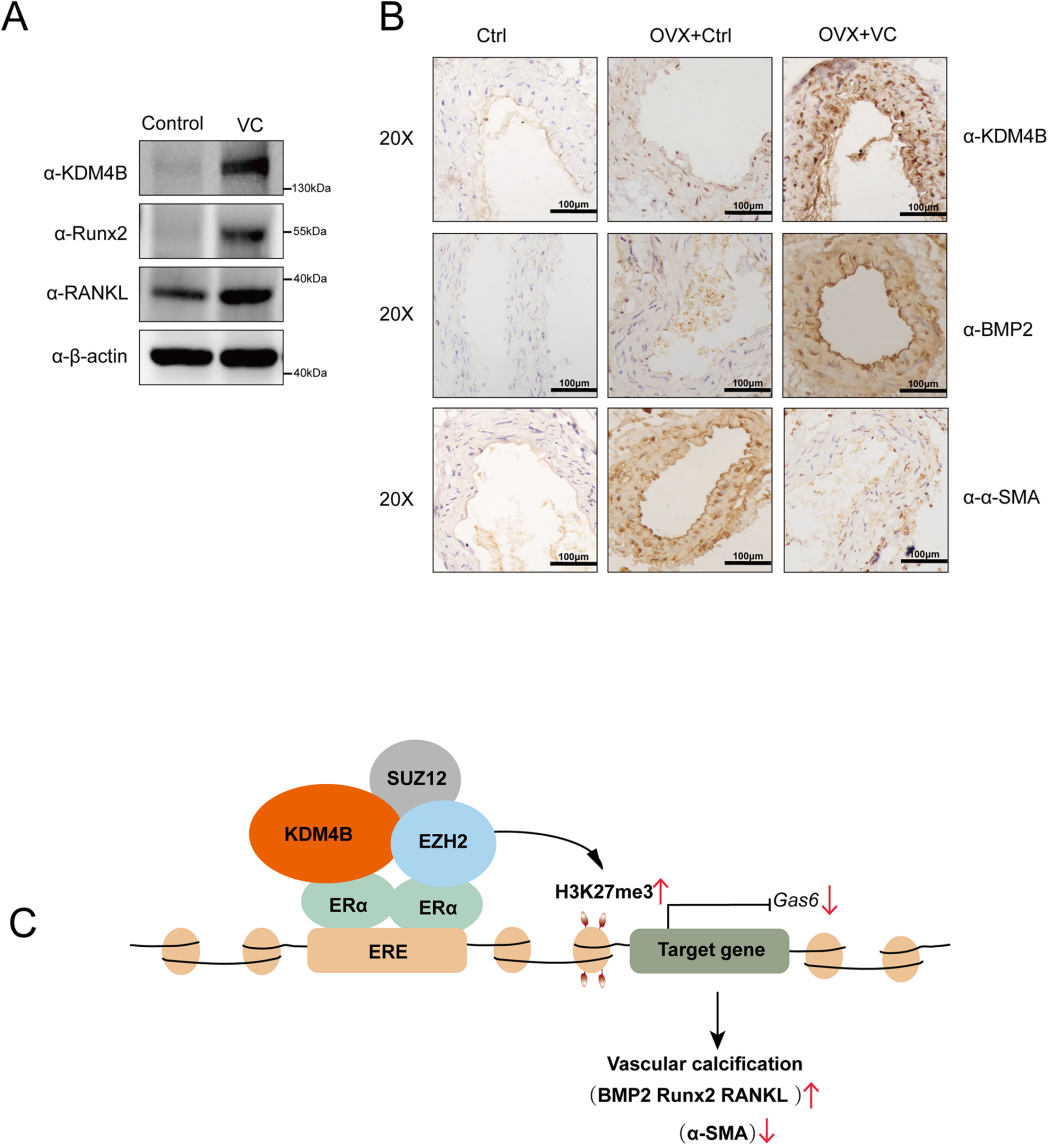
KDM4B is highly expressed in a VitD3-induced calcification mouse model. To construct a calcification model, WT and OVX mice were treated with or without 5 × 10^5^ UI/kg VitD3, respectively. **A** Western blot is comparing aortic protein levels (Runx2, RANKL) between mice treated with vitamin D3 vs. untreated controls. **B** Immunostaining results show that KDM4B and BMP2 show strong expression (brown staining) in calcified aortic sections from VitD₃-treated mice, whereas α-SMA (a smooth muscle marker) might be reduced or altered in those areas, indicating vascular smooth muscle cell changes during calcification. **C** A schematic summary of how KDM4B, as an ERα co-repressor, promotes vascular calcification. In estrogen-deficient conditions (such as OVX), elevated KDM4B associates with ERα and recruits PRC2 to repress protective genes (like Gas6), leading to increased Runx2/BMP2.

Ultimately, an in vivo calcification model was generated to confirm the in vitro findings. We generated a mouse calcification model using Vitamin D3 (Supplementary Fig. S5C–G). After the calcification protocol, we isolated the aortas and conducted western blotting and immunohistochemistry. The protein expression of KDM4B in the calcified aged mouse was higher than that in the control group (Fig. 8A). Immunohistochemical results showed that the KDM4B expression was enhanced in the ovary removal group (OVX) and further increased in the OVX with VitD3-induced calcification (OVX + VC) group. The expression levels of calcification marker BMP2 were increased, and the expression levels of α-SMA were decreased in the OVX + VC group (Fig. 8B). Our results suggest that KDM4B is highly expressed in VitD3-induced calcification mice.

## Discussion

In recent studies, the estrogen/ERα signaling pathway has been shown to play a significant role in VC. Estrogen inhibits the VC process in several ways, including the promotion of E2-induced gene transcription, promoting autophagy, and inhibiting the HIF-1α signaling pathway [8, 11]. ERα, as a typical transcription factor, mediates E2-induced gene transcription and inhibits VC. Co-activators and co-repressors of ERα are epigenetic enzymes and chromatin remodelers that activate or repress ERα-mediated gene transcription. KDM4B, as a histone demethylase, could regulate demethylation at histone H3K9 and H3K27. However, the epigenetic events responsible for the calcification process associated with KDM4B are mainly unknown. We, therefore, explored the mechanism underlying the function of KDM4B on the modulation of ERα-mediated transactivation in VC. Our study has shown that KDM4B suppresses ERα-mediated gene transcription independent of its demethylase activity. KDM4B interacts with the PRC2 complex to be recruited to the promoter region of the ERα target gene. In addition, KDM4B-mediated enhancement of ASMCs' calcification was partially attenuated by the estrogen treatment (Fig. 8C). Therefore, KDM4B may be a new potential therapeutic target for VC treatment.

It has been reported that the KDM4 family is a class of demethylases that can precisely remove methylation at H3K9 or H3K36 sites, thereby playing a central role in the histone code. A previous study demonstrated the relationship between KD M4B and ERα [45]. For example, KDM4B acts as an ERα cofactor that promotes mammary gland development and maturation. H3K9, KDM3A, and KDM4B synergistically regulate ERα activity through an autoregulatory loop that facilitates the recruitment of each co-activating enzyme to chromatin [45]. KDM4B recruitment within the upstream regulatory region of the ERα gene and demethylation of inhibitory H3K9me3 markers allow GATA-3 binding to drive receptor expression. This indicates the importance of KDM4B in the ERα signaling cascade and suggests a potential therapeutic target for BC therapy [40]. Deletion of KDM4B reduced WEE1, CCND1, and CCNA1 transcription and disrupted the destrier-induced cell cycle G1-S phase transition in BC cells, resulting in BC cell arrest in the G2-M or G1-S phase inhibition of BC occurrence [40, 41, 46]. We discovered that KDM4B overexpression promotes AR recruitment to the c-Myc gene enhancers and induces H3K9 demethylation; hence, increasing AR-dependent c-Myc mRNA transcription, which regulates sensitivity to next-generation AR-targeted therapies [47]. Although KDM4B enzymatic activity is required to enhance AR transcriptional activity, we have previously shown that KDM4B is an androgen-regulated demethylase that affects the transcriptional activity of AR not only through demethylation activity, but also through the regulation of ubiquitinylation [48]. KDM4B-regulated alternative splicing promotes AR-V7 expression and KDM4B promotes PCa cell growth via AR-V7 under androgen-deprivation conditions [49]. In our study, we used immunofluorescence to assess whether KDM4B and ERα co-localized in the nucleus in the presence or absence of calcification and E2. Co-IP results confirmed that they associated with each other in HASMCs and MOVAS cells. Interestingly, luciferase assay showed that KDM4B inhibited ERα-mediated gene transcription, whereas a mutant version of KDM4B with loss of demethylase activity (KDM4B HTE/ATA) had no synergistic effect on ERα in HASMCs and MOVAS cells. These results suggest that the down-regulation of ERα by KDM4B is independent of the enzymatic activity and is independent of the JmjC domain. Alternatively, KDM4B may bind to the original reader but not to another reader. The intricate chromatin environment may dictate the diverse functions exhibited by different tissues and various cell types.

Histone methyltransferase complexes consist primarily of histone methyltransferases and histone-interacting proteins that play a critical role in chromatin remodeling, histone modification, cell differentiation, or tumorigenesis in mammalian cells [50]. The PRC2 core subcomplex has H3K27 methyltransferase activity, which contains EZH2, SUZ12, and other potentially combinatorial proteins [50–54]. The STRING database was used to predict the proteins that may be related to KDM4B, and we discovered that KDM4B may interact with EZH2 and SUZ12 in the presence of ESR1 (ERα). In HASMCs and MOVAS cells, we provide evidence that KDM4B could interact with the PRC2 subcomplex alongside ERα, thereby influencing the spatial interaction between PRC2 proteins and ERα. On chromatin, KDM4B can affect the recruitment of PRC2 complexes to specific DNA regions. In addition, KDM4B promoted the recruitment of PRC2 complex core proteins and further altered the histone modification level in the promoter regions of ERα target genes. These results suggest that KDM4B, an ERα co-regulator and a PRC2 complex bridge protein, might be involved in complex chromatin events essential for epigenetic modulation of ERα-mediated gene transcription in VC.

It has been reported that KDM4B plays a crucial role during bone formation. KDM4B further enhances the expression of DLX2 and DLX5 by removing H3K9me3 marks; hence, facilitating osteogenic commitment [55, 56]. In the process of osteoclast formation, KDM4B physically binds to CCAR1-MED1 to form a complex, which localizes to the promoter of multiple osteoclast-related genes and alters the chromatin structure through the action of histone demethylase, thereby promoting the expression of related genes [57]. Moreover, although it has also been shown that KDM4B knockdown weakened the osteogenic differentiation in MSCS, the audiogenic differentiation was enhanced. Loss of KDM4B increases H3K9me3, which reduces bone formation and increases bone marrow fat, thereby exacerbating bone aging and osteoporosis [58]. In our study, KDM4B was highly expressed in the calcification model, and we showed it played a role in modulating ERα-mediated gene transcription regulation. Ectopic expression of KDM4B aggravated mineralization and increased calcium content as well as the expression levels of calcification markers (BMP2, Runx2, and RANKL); thus, indicating that calcification is exacerbated by KDM4B. Additionally, KDM4B-mediated enhancement of β-GP-induced calcification was attenuated by the estrogen treatment. This suggests that E2/ERα underlies the function of KDM4B in VC. Our data have revealed that KDM4B acts as an ERα-mediated transcriptional regulator independent of its demethylase activity. Currently, VC is considered an independent predictor of cardiovascular events. For example, simple supplementation with estrogen might alleviate VC; however, such treatment might be associated with numerous estrogen-specific adverse effects and might not be conducive to treating diseases [10, 59, 60]. Our findings uncover an epigenetic factor that acts as a novel co-regulator of ERα, participating in the VC pathway. This discovery offers vital insights for postponing the onset of cardiovascular disease in older adults. Furthermore, our findings indicate that KDM4B could be utilized as a marker for predicting VC and might even emerge as a viable therapeutic target for treating this condition.

Our study has demonstrated that KDM4B is highly expressed in β-GP-induced HASMCs and MOVAS cell calcification models. KDM4B co-represses ERα-induced transactivation independent of its demethylase activity in HASMCs and MOVAS cells. Furthermore, it facilitates the recruitment of PRC2 core proteins to the cis-regulatory elements of ERα target genes. Therefore, we believe that KDM4B is a potential therapeutic target for calcification, providing a novel insight into clinical treatment.

## Experimental procedures

### Cell line culture and in vitro calcification model

HASMCs and MOVAS cells were cultured in Dulbecco’s modified Eagle medium (DMEM, SERAXPRO). The HEK-293 cell was cultured in DMEM (SERAXPRO). Cells were cultured with 10% fetal bovine serum (FBS, SERAXPRO), 100 U/mL penicillin, and 100 μg/mL streptomycin (Glenview, Florida, USA) in a 37 °C incubator with a humidified, under 5% CO_2_ atmosphere. Next, 17-estradiol (E2, Sigma–Aldrich, St. Louis, MO, USA) was dissolved in ethanol (Aladdin Scientific, Riverside, CA, USA). Cells were cultured in phenol-red-free DMEM or RMPI1640 when needed to add an E2 stimulus. Culture dishes were purchased from Guangzhou Jet Bio-Filtration Co., Ltd., China. When VSMC reaches 50~70% confluency, VSMCs are treated with calcification medium containing 1% FBS and 5 mM β-GP. The calcification medium was changed every 2 days to induce calcification in VSMC.

### Antibodies

The following antibodies were used in this study: anti-KDM4B (Cell Signaling Technology, Danvers, MA, USA, #8639), anti-KDM4B (ABclonal, Woburn, MA, USA, #A6670), anti-ERα (Cell Signaling Technology, #D8H8), anti-Gas6 (Proteintech, Sankt-Leon Rot, Germany, #13795-1-AP), anti-β-actin (Proteintech, #66009-1-AP), anti-EZH2 (Proteintech, #21800-1-AP), anti-SUZ12 (Cell Signaling Technology, #3737), anti-H3K9me3 (Cell Signaling Technology, #13969) and anti-H3K27me3 (Cell Signaling Technology, #9733), anti-Rabbit/Mouse (ABclonal), anti-IgG (Proteintech, #10238-1-AP), anti-HA (ABclonal M20003M), BMP2 (Proteintech, #18933-1-AP), anti-Runx2 (Sigma, #AV36678), and anti-RANKL (Proteintech, #23408-1-AP).

### Transfections, siRNA, and luciferase dual reporter assay

The HA-KDM4B plasmid was purchased from Addgene (Plasmid #24181). According to a previous study, the HA-KDM4B mutant was cloned into the PCMV-HA vector [44]. Expression plasmids for ERα, ERα-AF1, and ERα-AF2 were kindly provided by Dr Shigeaki Kato [61]. Final constructs were verified using DNA sequencing. siRNA against KDM4B was purchased from JTS Scientific (London, UK). All sequences are listed in Supplementary Table S1.

Cell lines were co-transfected with the listed constructs according to the manufacturer’s instructions using jetPRIME^TM^ DNA Transfection Reagent (Polyplus transfection). KDM4B and its truncated mutants, ERα, and ERE-tk-Luc were co-transfected with a plasmid containing Renilla luciferase (pRL). After co-transfection, cells are cultured in medium containing 3% charcoal-stripped fetal bovine serum (CS-FBS) for 6 h with or without E2 stimulation. After another day, cells were collected for dual-luciferase reporter assay (Promega, Madison, WI, USA).

### Western blotting analysis, Co-immunoprecipitation, and glutathione sepharose (GST) pull-down assays

Western blotting analysis was performed using a standard procedure introduced in previous studies [62, 63]. In brief, cells or vascular tissues were lysed on ice for 30 min using lysis buffer [50 mM Tris/HCl (pH 7.4), 150 mM NaCl, 1% NP‑40, 1% Triton X-100, 0.25% sodium deoxycholate, 1 mM EDTA, and protease inhibitor cocktail (B14001, Houston, TX, USA)]. Lysates were vortexed every 10 min and then centrifuged at 13,000 × *g* for 20 min at 4 °C. Next, the supernatant protein contents were measured using G250, and 30–50 μg lysates were prepared for blotting. Samples were loaded into 8% or 12% polyacrylamide gels, separated by SDS-PAGE, and transferred onto PVDF membranes (ISEQ00010, Millipore, Burlington, MA, USA) under 80 v for 120 min. Next, membranes were blocked with 5% nonfat milk in TBST solution [20 mM Tris (pH 7.4), 137 mM NaCl, and 0.05% Tween‑20] for 1 h at room temperature and probed with primary antibodies overnight at 4 °C. The next day, after washing the membranes with TBST solution three times, the membranes were incubated with secondary antibodies for 1 h at room temperature. Finally, the membranes were detected by chemiluminescence.

Co-immunoprecipitation begins with whole-cell lysis after 2 h of purification with anti-IgG. Protein G beads were purchased from GE HealthCare (Chicago, IL, USA) and concentrated into sepharose for overnight rotation of antibody-protein interactions.

The GST pull-down assay was performed using a standard protocol introduced in a previous study [62]. GST alone proteins and GST fusion proteins, including GST-ERα 29-180aa and GST-ERα 282-595aa, were expressed in BL21 and bound to GST-sepharose beads according to the manufacturer’s instructions (GE HealthCare). The HA-KDM4B expression plasmid was constructed to synthesize the HA-KDM4B protein. Equal amounts of GST alone or conjugated GST-sepharose beads of GST fusion protein with in vitro translated HA-KDM4B protein were incubated overnight at 4 °C. The precipitated proteins were washed 4 times with binding buffer (20 mM Tris, pH 7.5, 50 mM NaCl, 10% glycerol, and 1% Nonidet P-40). Western blotting and Coomassie brilliant blue staining were used to detect binding proteins.

### Confocal immunofluorescence imaging

After 48 h of treatment under the specified conditions (with or without 100 nM E2 in the presence of β-glycerophosphate to initiate calcification), cells were fixed in 4% paraformaldehyde for 15 min at room temperature. The fixed cells were then blocked with 1% donkey serum albumin to prevent nonspecific binding. Next, cells were incubated with primary antibodies overnight at 4 °C. After thorough washing, the cells were incubated with secondary antibodies conjugated to FITC, Cy3, or Cy5 (Jackson Immunoresearch Laboratories Inc., West Grove, PA, USA) for signal detection. Finally, nuclei were counterstained with DAPI (Roche, Zurich, Switzerland) to visualize nuclear morphology. Fluorescent signals were observed and imaged using a confocal microscope.

### RNA and quantitative real-time PCR (qPCR)

After transfection of control and KDM4B siRNA, RNA was extracted. At 4–6 h after transfection, estrogen and the same amount of EtOH were added to a final concentration of 100 nM for 16–18 h. Total RNA was extracted with RNA Trizol (TAKARA Bio, Tokyo, Japan), and the PimeScript RT-PCR Kit (TAKARA Bio) was used to reverse transcribe the cDNA. The SYBR premeraseTaq Kit (TAKARA Bio) was used for real-time PCR detection of Roche’s LightCycler96. All primers for qPCR are listed in Supplementary Table S2. Statistical analysis was performed using PRISM GraphPad 8. Each experiment was performed at least in triplicate, and significance was tested using Student’s *t*-tests.

### Chromatin immunoprecipitation (ChIP)

ChIP assays were performed using a standard protocol [64]. HASMCs were transfected with HA-tagged plasmids to overexpress KDM4B or KDM4B siRNA to knock down its expression. After transfection, cells were cultured in phenol-red DMEM medium containing 10% CS-FBS for 2 days. When the cell confluency reached 80%, cells were stimulated with 100 nM E2 or an equivalent amount of EtOH for 12 h. After the experiment, qPCR was performed using DNA as the template (primers are listed in Supplement Table S3).

### Alizarin red staining

Cells were washed thrice with PBS solution, fixed with 4% paraformaldehyde (#AR-0211, Dingguo Changsheng Biotechnology Co., Ltd., Beijing, China) for 30 min at room temperature, then washed with PBS solution and stained with 1% Alizarin red (pH 4.2, #G1452, Solarbio, Beijing, China) for 30 min at room temperature. Finally, stained cells were rinsed 3 times with distilled water at room temperature and imaged.

### Quantification of calcium deposition

Cells or the vascular tissues were washed with PBS solution and then decalcified with 0.6 M HCL at 37 °C for 24 h. The supernatant was collected and used for calcium quantification (Biosino Bio-Technology And Science Inc., Beijing, China). Next, cells or tissues were lysed (0.1 M NaOH, 0.1% SDS) for 30 min, then centrifuged at 12,000 × *g* for 20 min at 4 °C, and the protein concentration was measured using a BCA kit (Cat NO: KGP903, Keygen Biotech, Nanjing, China) to normalize the calcium levels.

### Animal calcification model

Mice were brought from Beijing Vitonglihua Experimental Animal Technology Limited Company (China). All experiments were approved by the Institutional Animal Protection and Use Committee (IACUC) of China Medical University and performed in accordance with the IACUC guidelines. To induce VC phenotypes in vivo, we used classical and recognized mouse calcification models. Mice were injected subcutaneously with VitD3 solution [5 × 10^5^ UI/kg VitD3 mixed in corn oil], weighed once every 3 days, and sacrificed on the 7th day.

### Whole-mount Alizarin Red S staining (ARS)

Mice aortas were first fixed in 4% paraformaldehyde (PFA) for 24–48 h. The fixed aortas were then fully immersed in 95% ethanol at room temperature for 12 h to dehydrate the tissue. After dehydration, aortas were transferred to a 0.004% Alizarin Red S solution (prepared in 1% KOH) and incubated at room temperature for 12 h (overnight). The next day, the aortas were washed with 2% KOH (at least twice) to remove residual stain until non-calcified areas appeared milky white. The staining intensity and extent of calcification in the aortas were compared between different groups, and photographs were taken to document the results. Finally, the aortas were stored in 4% PFA at room temperature for long-term preservation.

### Alizarin Red S staining of tissue sections

Rehydrated vascular tissue sections were stained with 2% Alizarin Red S (pH 4.2) at room temperature for 15–30 min. After staining, sections were rinsed under gently running distilled water until the rinse water became nearly colorless. Care was taken to avoid directing the water stream straight onto the tissue sections during washing to prevent dislodging the samples. The slides were then examined under a microscope to observe the Alizarin Red S staining.

### Von Kossa staining

Vascular tissue sections were placed in 1% silver nitrate solution and exposed to ultraviolet light (with a piece of foil or mirror positioned behind the slides to reflect light) for 60 min. After this silver nitrate treatment, sections were rinsed in distilled water for 5 min, then immersed in 5% sodium thiosulfate for 2 min to neutralize any remaining silver, followed by another 5 min rinse in distilled water. Next, the sections were counterstained with 1% basic fuchsin for 1–2 min and briefly rinsed with distilled water for 5–10 s. The staining results were observed under a microscope.

### Statistical analysis

All results are presented as the mean ± standard deviation (SD), and analyses were performed using the PRISM GraphPad 8 software program. A two-sided Student’s *t*-test was used to determine the significant differences in calcium deposition, the real-time PCR data, and the luciferase assay. Significance was measured using *p*-values (*p < 0.05, **p < 0.01, and ***p < 0.001).

## Supplementary information

Supplementary Table

Supplementary Figure

Original Data File

## Data Availability

All data generated or analyzed during this study are included in this published article and its supplementary information files.
