# Supplementary material for: KDM4B modulates ERα signaling pathway to participate in vascular smooth muscle cell calcification
Source: Cell Death Discov. 2025 Oct 7;11:452. doi: 10.1038/s41420-025-02765-6 (PMC12504744; doi:10.1038/s41420-025-02765-6)
Supplement: Supplementary file 1 — Supplementary Table [file 41420_2025_2765_MOESM1_ESM.docx]

**Supporting Information**

**KDM4B modulates ERα signaling pathway to participate**

**in vascular smooth muscle cell calcification**

Fei Liu^1, 3^, Yang Lv^1^, Yanxia Lin^1^, Chunyu Wang^2^, Shengli Wang^2^, Kai Zeng^2^, Baosheng Zhou^2^, Lin Lin^2^, Jianwei Feng^2^, Ge Sun^2^, Xiaocen Chang^2^, Mengsu Cao^2^, Hao Li^2^, Xihong Hu^3^, Kato Shigeaki^4,5^, Yue Zhao^2^*, Wen Tian^1^*

^1^ Department of Geriatric, the First Affiliated Hospital of China Medical University, Shenyang City 110001, Liaoning Province, China.

^2^ Department of Cell Biology, Key Laboratory of Medical Cell Biology, Ministry of Education, School of Life Sciences, China Medical University, Shenyang City 110122, Liaoning Province, China.

^3^ Department of Geriatric, Dalian Friendship Hospital Affiliated to Dalian Medical University.

^4^Graduate School of Life Science and Engineering, Iryo Sosei University, Iino, Chuo-dai, Iwaki, Fukushima 9708551, Japan.

^5^Research Institute of Innovative Medicine, Tokiwa Foundation, Iwaki, Fukushima, Japan.

To whom correspondence should be addressed.

1. Wen Tian. Department of Geriatric, The First Affiliated Hospital of China Medical University, No.155 Nanjing North Street, He Ping District, Shenyang City 110001, Liaoning Province, China. Tel: +86 24 83283333;

email: dr_wentian@163.com

2. Yue Zhao. Department of Cell Biology, Key Laboratory of Medical Cell Biology, Ministry of Education, School of Life Sciences, China Medical University, No.77 Puhe Road, Shenyang North New Area, Shenyang City 110122, Liaoning Province, China.

Tel: +86 24 31939077; Fax: +86 24 31939077;

email: y[zhao30@cmu.edu.cn](mailto:zhao30@cmu.edu.cn)

**Supplemental Materials, including:**

**1. Supplementary data**

**2. Supplementary Figure Legends**

**Supplemental Figure S1.** KDM4B is highly expressed in β-GP induced calcification models in HASMCs.

**Supplemental Figure S2.** KDM4B regulate ERα-induced transactivation independent on its demethylase activity in MOVAS cells.

**Supplemental Figure S3.** KDM4B modulate on the expression of estrogen-inducing gene mRNA and protein in MOVAS cells.

**Supplemental Figure S4.** Ectopic expression of KDM4B aggravates β-GP induced calcification in HASMCs.

**Supplemental Figure S5.** KDM4B-mediated enhancement of β-GP induced calcification is attenuated by the treatment of estrogen and Vitamin D₃-Induced Aortic Calcification in OVX Mice.

**Supplementary table 1. siRNA used in this study**

| Name | Sense(F’) | Anti-sense(R’) |
| --- | --- | --- |
| KDM4B#1 | CCUCUUCACGCAGUACAAUTT | AUUGUACUGCGUGAAGAGGTT |
| KDM4B#3 | GCCGGAAGCGGAUGAAGAATT | UUCUUCAUCCGCUUCCGGCTT |

dTdT: DNA bases within RNA oligos.

**Supplementary table 2. Primers used for quantitative RT-PCR in HASMC**

| Name | Sense(F’) | Anti-sense(R’) |
| --- | --- | --- |
| KDM4B | CGGGTTCTATCTTTGTTTCTCTCACCCG | AAGGAAGCCTCTGGAACACCTG |
| BMP2 | ACCCGCTGTCTTCTAGCGT | TTTCAGGCCGAACATGCTGAG |
| Runx2 | ATCCCCATCCATCCACTCCA | GGGGTGTAGGTAAAGGTGGC |
| α-SMA | GTACCACCATGTACCCAGGC | GCTGGAAGGTAGACAGCGAA |
| ESR1 | CTAACTTGCTCTTGGACAGGAA | CAGGACTCGGTGGATATGGT |
| Gas6 | CATCAACAAGTATGGGTCTCCGT | GTTCTCCTGGCTGCATTCGTTGA |
| β-actin | CTCGCCTTTGCCGATCC | GGGGTACTTCAGGGTGAGGA |

**Supplementary table 3. Primers of ChIP used for quantitative RT-PCR**

| Name | Sense(F’) | Anti-sense(R’) |
| --- | --- | --- |
| Gas6-ERE | CAGACCGAGCGCTTGAGG | GAGAGCGAAGGGGCCAT |
|  |  |  |
