# Supplementary material for: KDM4B modulates ERα signaling pathway to participate in vascular smooth muscle cell calcification
Source: Cell Death Discov. 2025 Oct 7;11:452. doi: 10.1038/s41420-025-02765-6 (PMC12504744; doi:10.1038/s41420-025-02765-6)
Supplement: Supplementary file 2 — Supplementary Figure [file 41420_2025_2765_MOESM2_ESM.docx]

**Supplementary Figures**


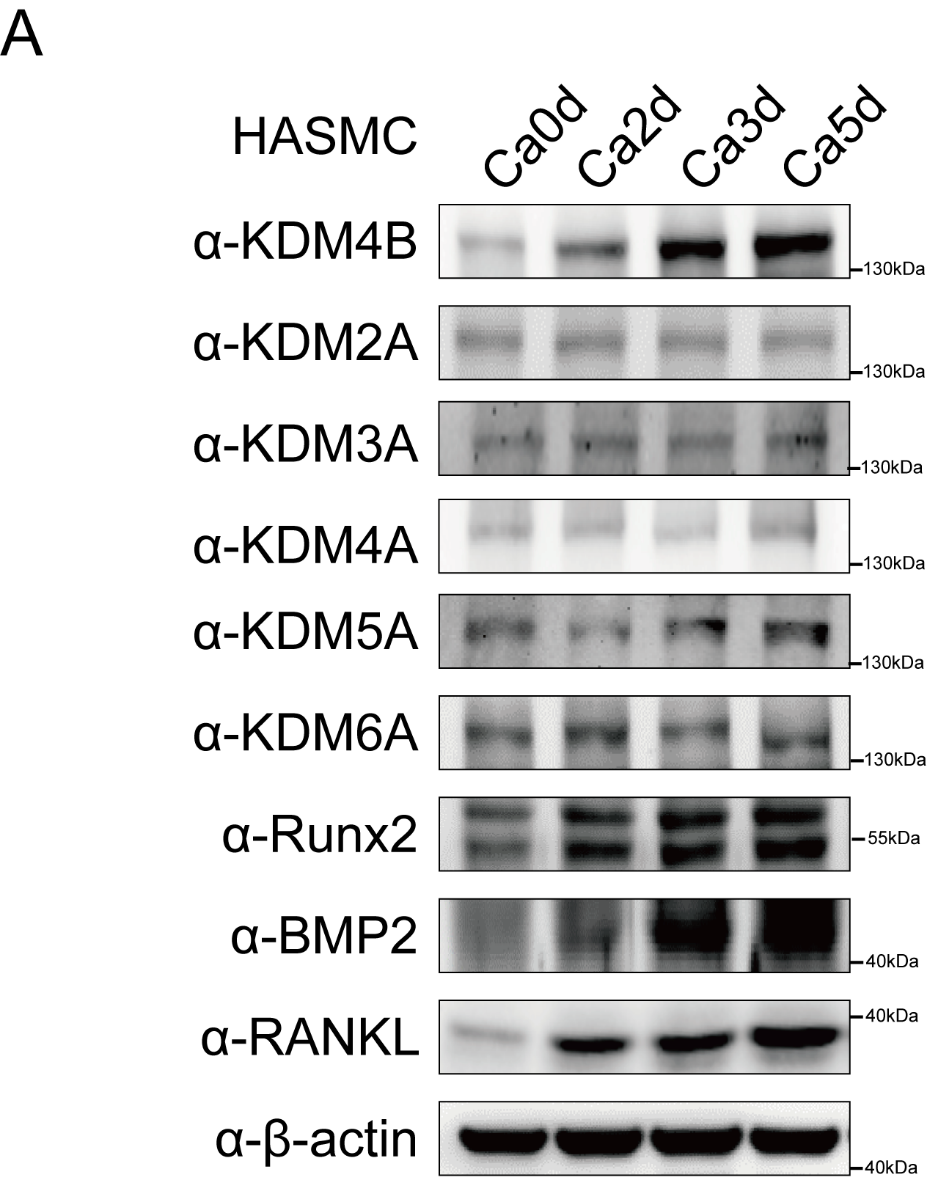


**Supplemental Figure S1**

**Figure S1. KDM4B is highly expressed in β-GP induced calcification models in HASMCs.**

(A)Western blot analysis for KDM4B, KDM2A, KDM3A, KDM4A, KDM5A, KDM6A, Runx2, BMP2, RANKL protein expression in different days in HASMCs treated with 5mM β-GP, β-actin was used as a loading control, and the day 0 was set as the control in each parameter. **p* < 0.05, ** *p* < 0.01 and ns stands for no significance.


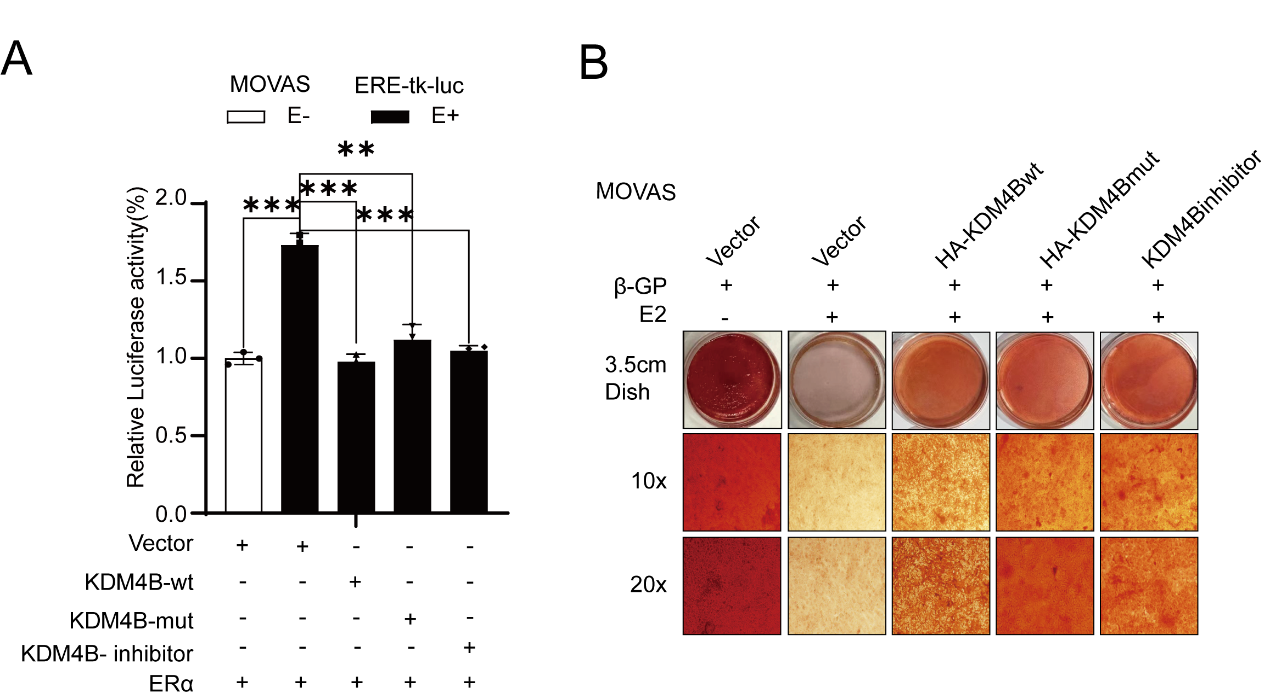


**Supplemental Figure S2**

**Figure S2 KDM4B regulate ERα-induced transactivation independent on its demethylase activity in MOVAS cells.**

(A) KDM4B (HTE/ATA) and KDM4B inhibitor carrying a loss of function in demethylase activity exhibits reduced coactivation function on ERα action in MOVAS cells. (B) Alizarin red staining in MOVAS cells. KDM4Bwt overexpression, KDM4Bmut overexpression and KDM4B inhibitor-mediated enhancement of β-GP induced calcification in MOVAS cells.


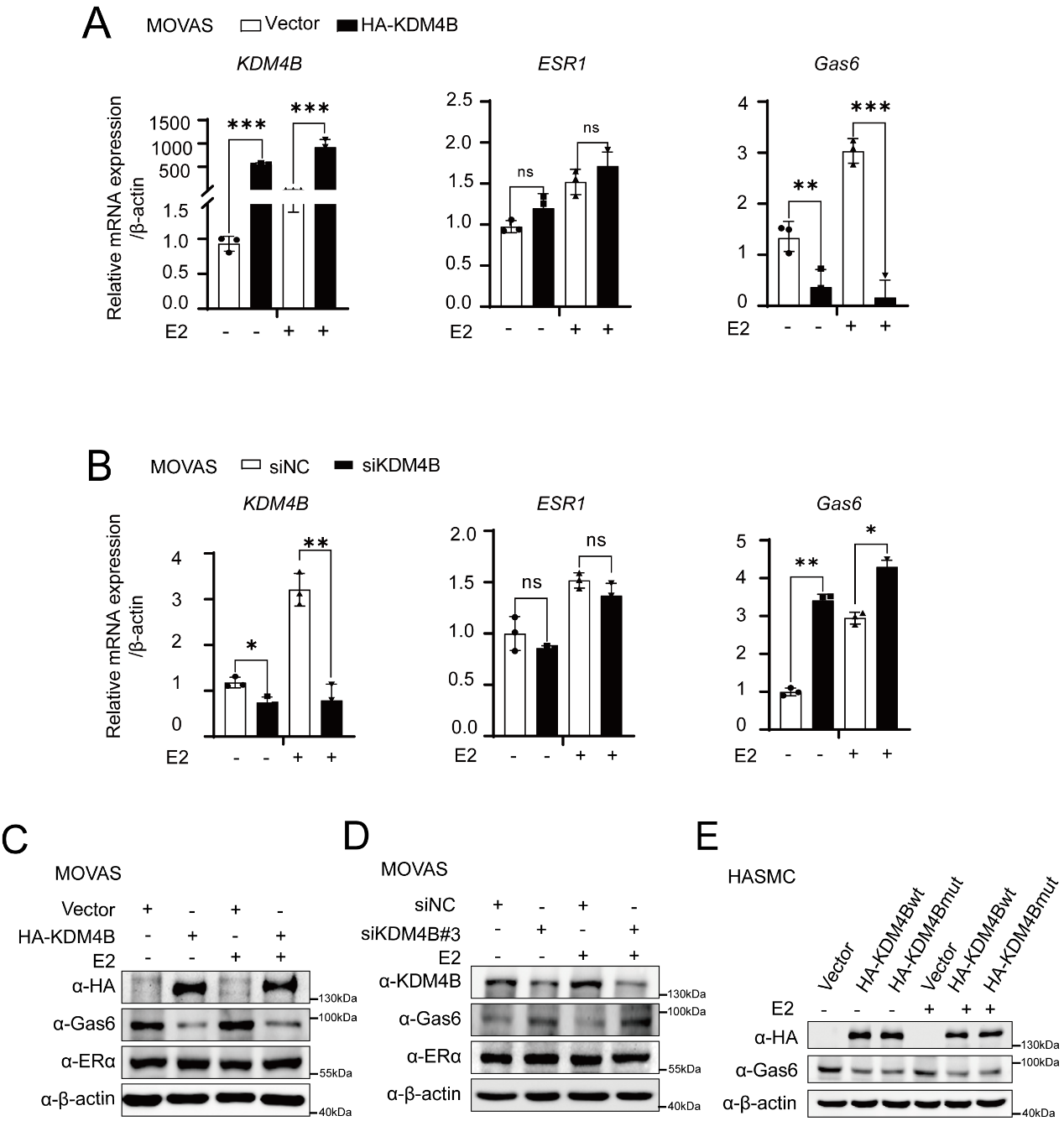


**Supplemental Figure S3**

**Figure S3 KDM4B modulate on the expression of estrogen-inducing gene mRNA in MOVAS cells.**

(A-B) qPCR analysis was examined for the effects of KDM4B on mRNA expression of endogenous ERα target genes in MOVAS cells. Statistical significance was determined using Student t-tests. Error bars represent mean± SD. **p* < 0.05, ** *p* < 0.01 and ns stands for no significance. (C-D) The effects of KDM4B on protein expression of endogenous ERα target genes as indicated in MOVAS cells. β-actin was used as a control. (E) The effects of KDM4Bwt and KDM4B mutant on protein expression of endogenous ERα target genes as indicated in HASMCs. β-actin was used as a control.


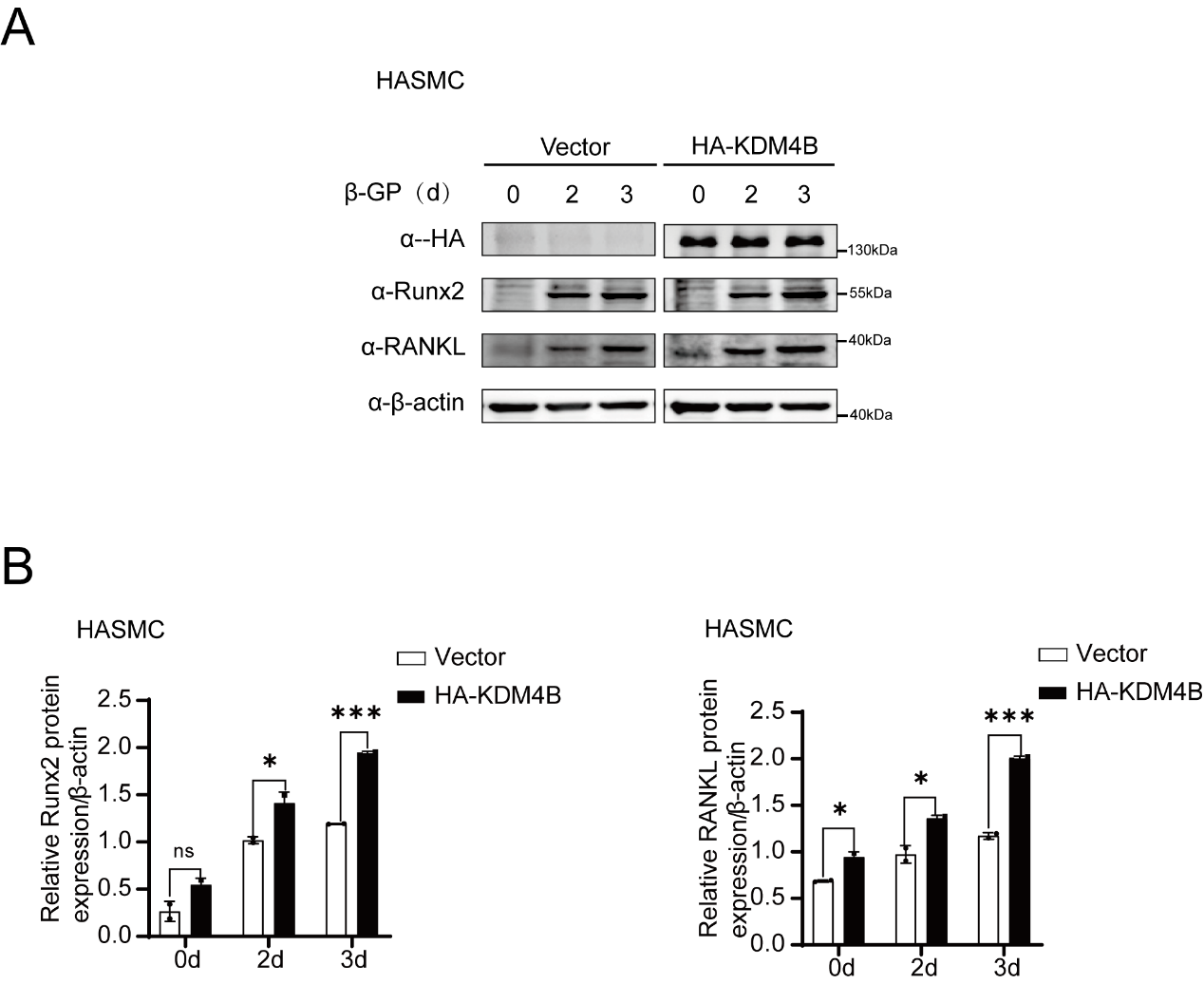


**Supplemental Figure S4**

**Figure S4 Ectopic expression of KDM4B aggravates β-GP induced calcification in HASMCs.**

(A-B) Western blot analysis and quantification of protein expression for HA-KDM4B, Runx2, RANKL and β-actin in HASMCs, with treated by β-GP was set as control in each parameter. Data were expressed as the mean ± SD of triplicate experiments. **p* < 0.05, ** *p* < 0.01 and ns stands for no significance.


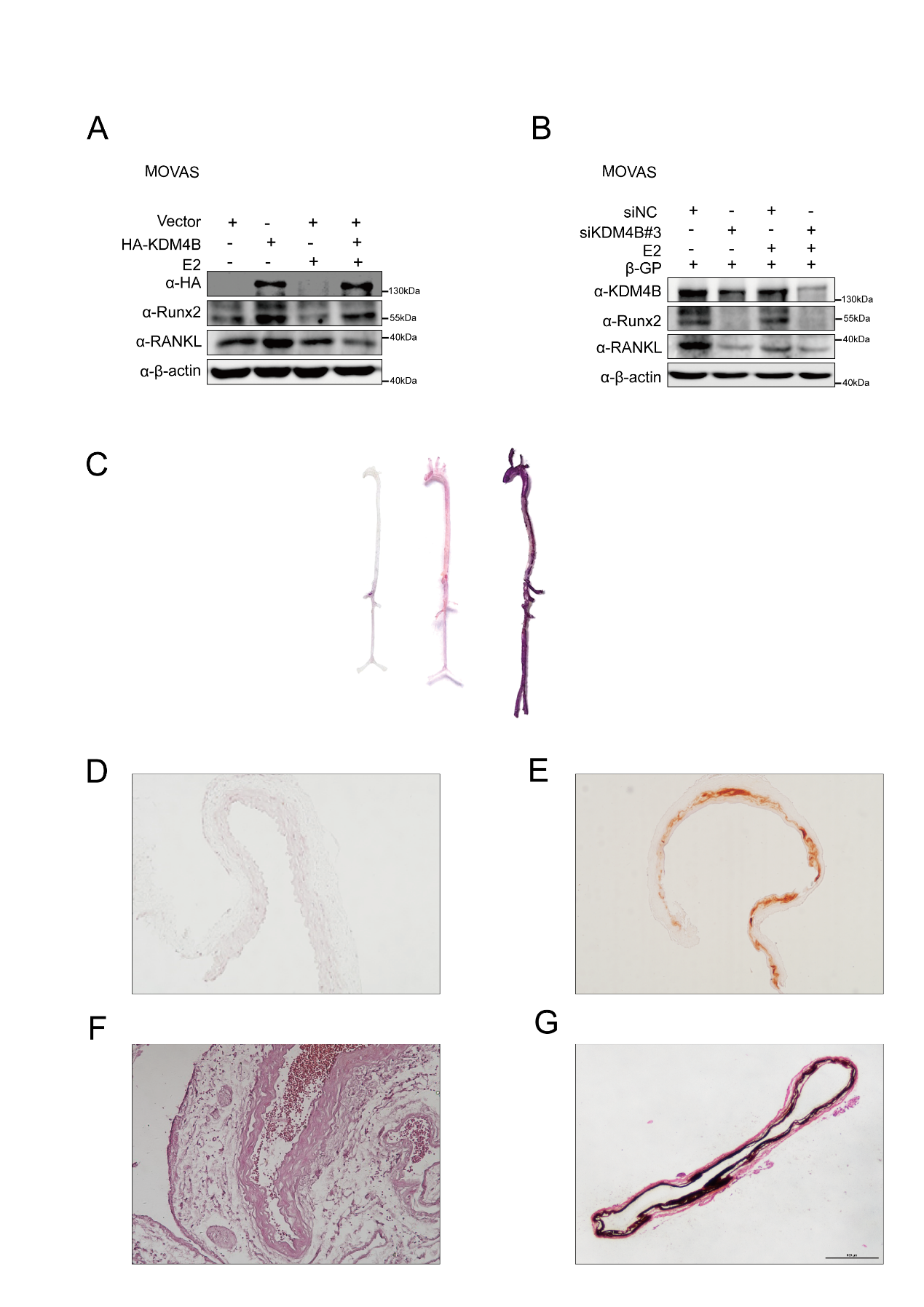


**Supplemental Figure S5**

**Figure S5 KDM4B-mediated enhancement of β-GP induced calcification is attenuated by the treatment of estrogen** **and** **Vitamin D₃-Induced Aortic Calcification in OVX Mice.**

(A) Western blot assay indicated the protein expression for HA-KDM4B, Runx2, RANKL and β-actin, β-actin was used as a loading control. (B)The KDM4B depletion by siRNA against KDM4B (siKDM4B) in MOVAS cells and with or without treated by estrogen was set as the control in each parameter. Data were expressed as the mean ± SD of triplicate experiments. (C) Show alizarin red staining of the whole mouse aortic tree in WT and OVX mice with and without Vit D treatment. (D, E) Alizarin red staining of the aortic cross sections. OVX+ Ctrl vs. OVX+VC (100x). (F, G) Von Kossa of the aortic cross sections. OVX+ Ctrl vs. OVX+VC (100x).
