# Supplementary figures and images for: KDM4B modulates ERα signaling pathway to participate in vascular smooth muscle cell calcification
Source: Cell Death Discov. 2025 Oct 7;11:452. doi: 10.1038/s41420-025-02765-6 (PMC12504744; doi:10.1038/s41420-025-02765-6)

Fig 1

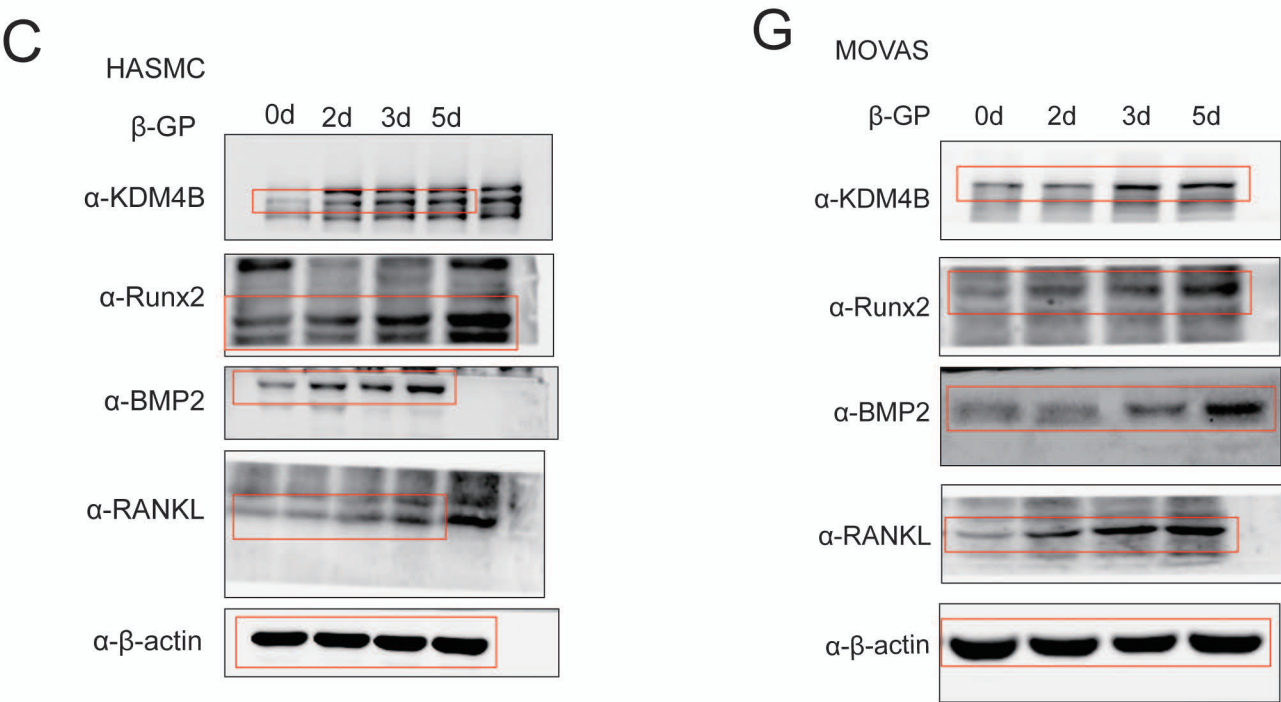

Fig 2

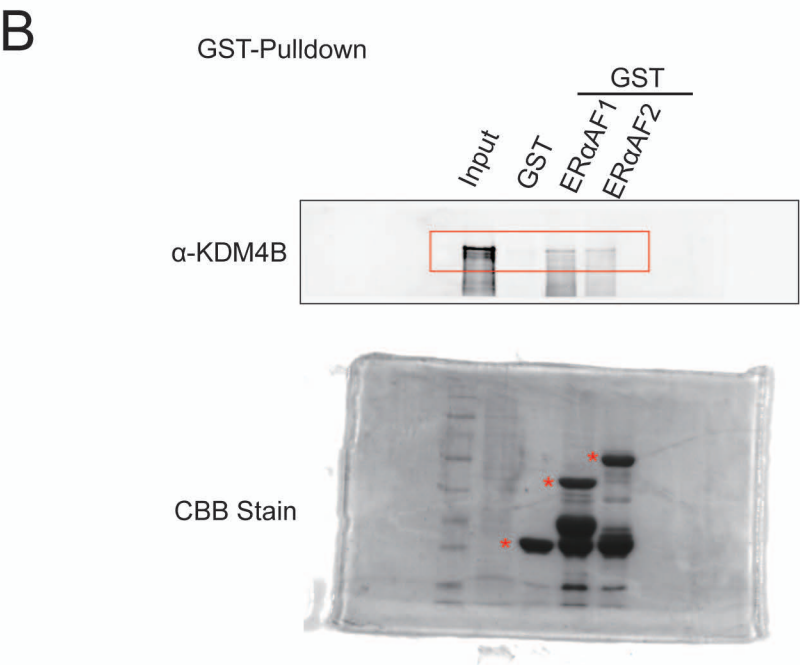

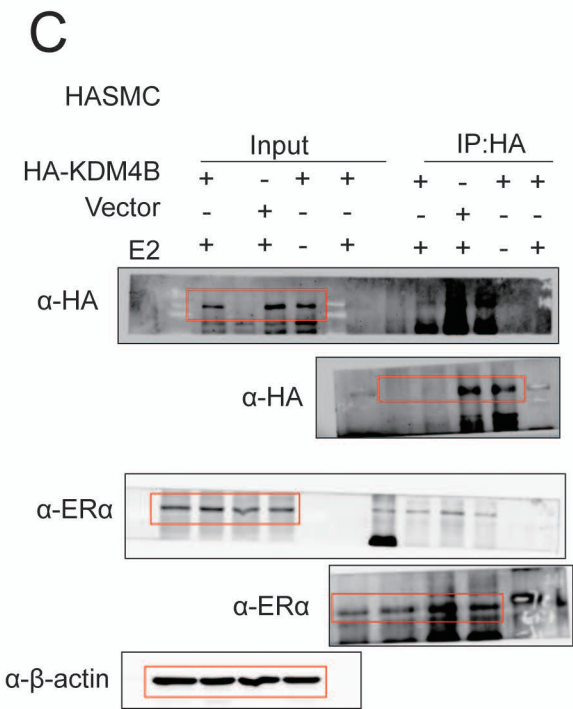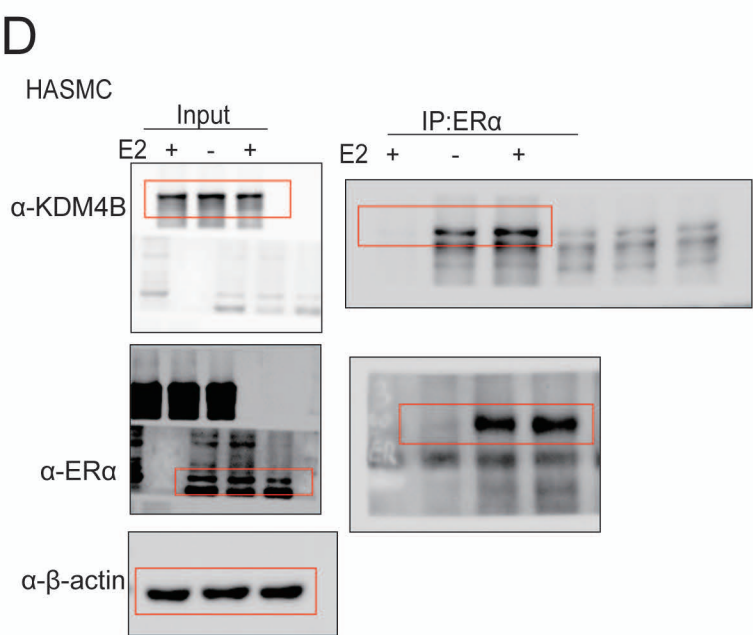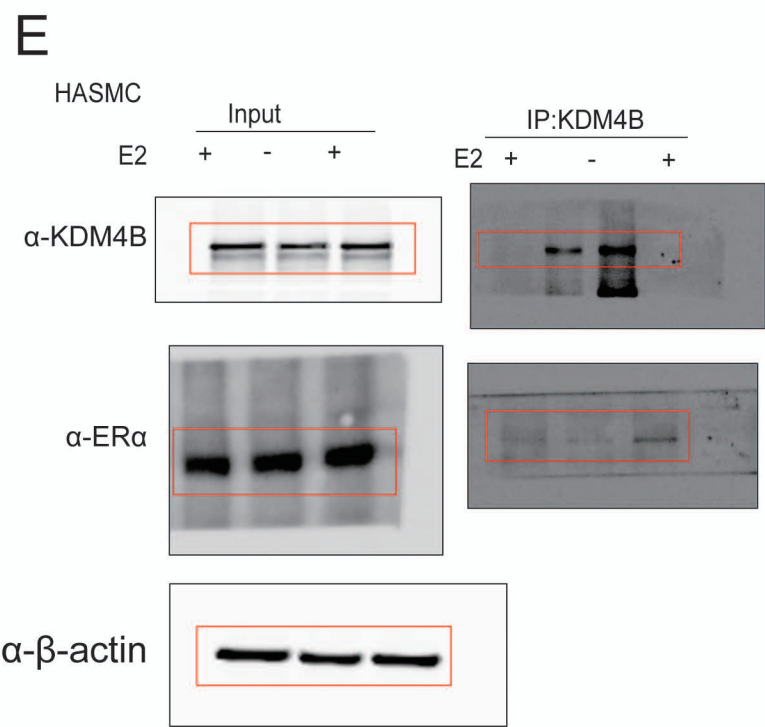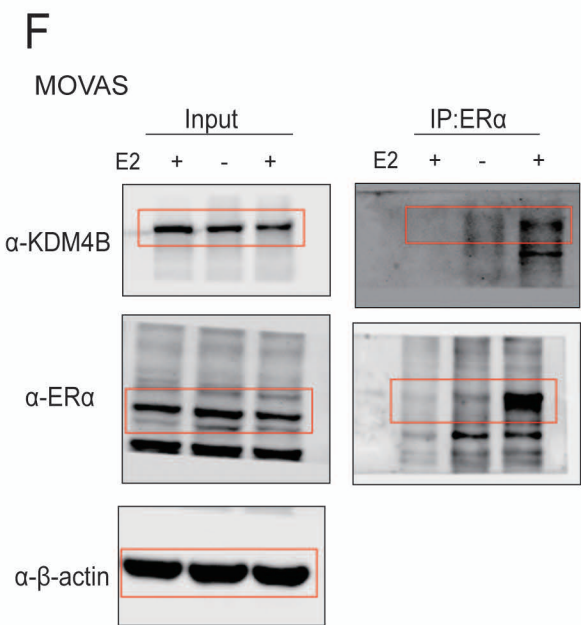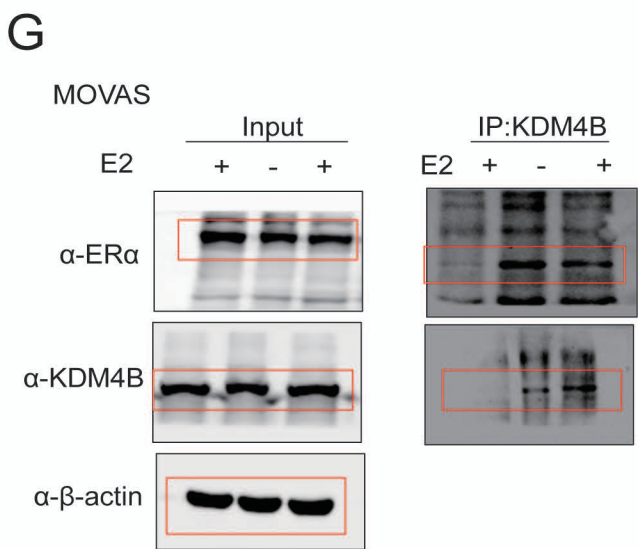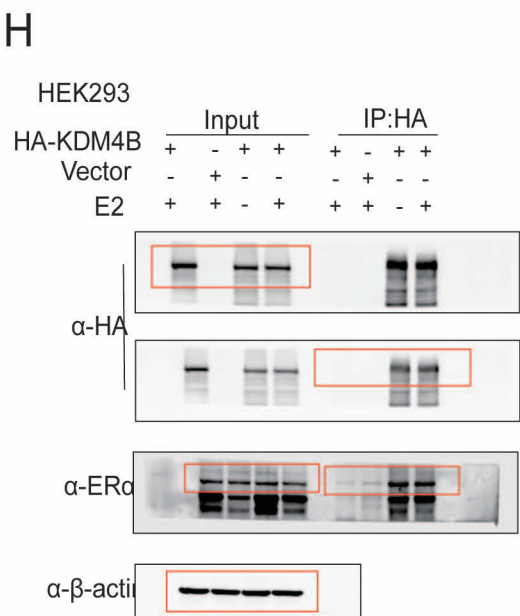

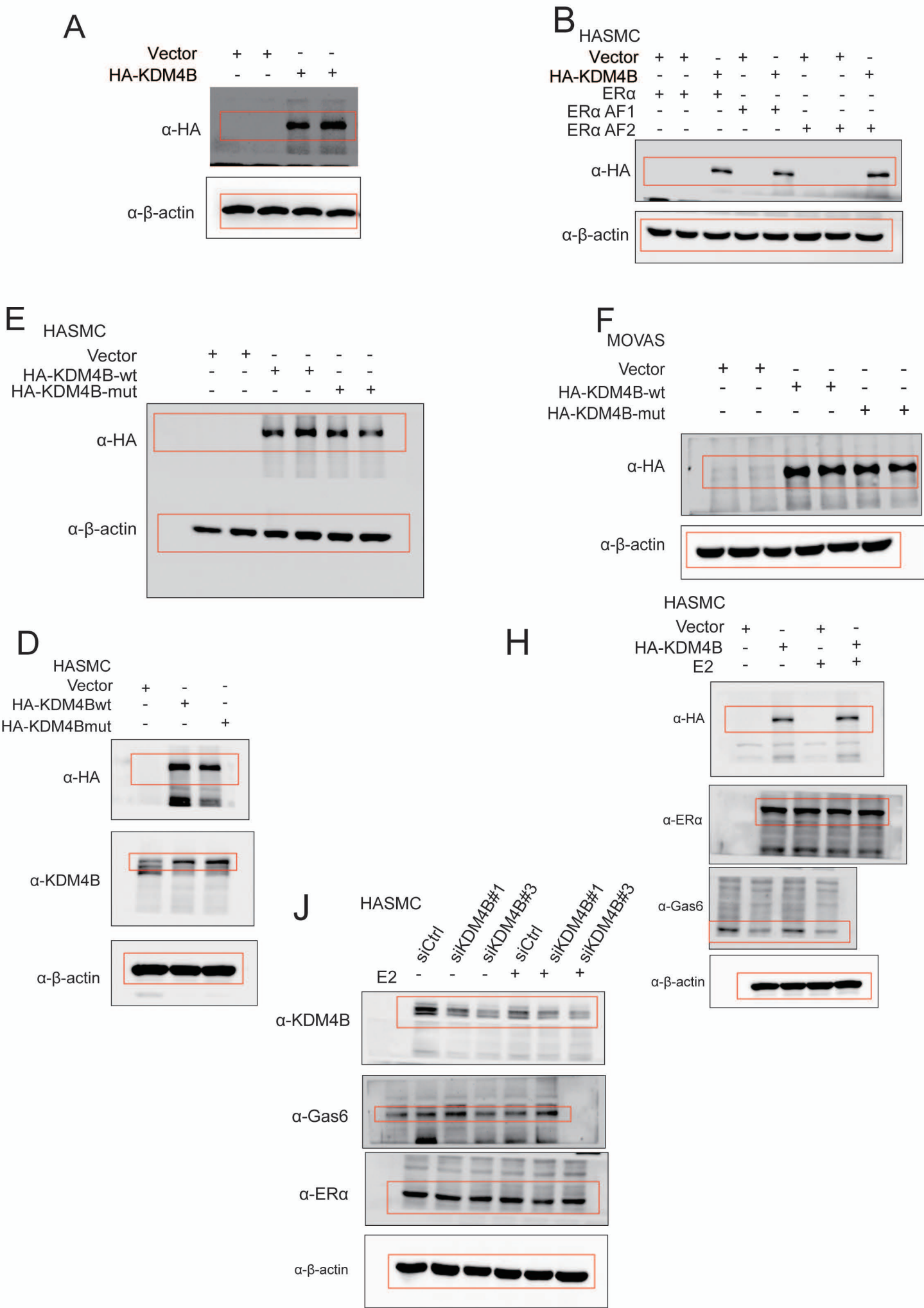

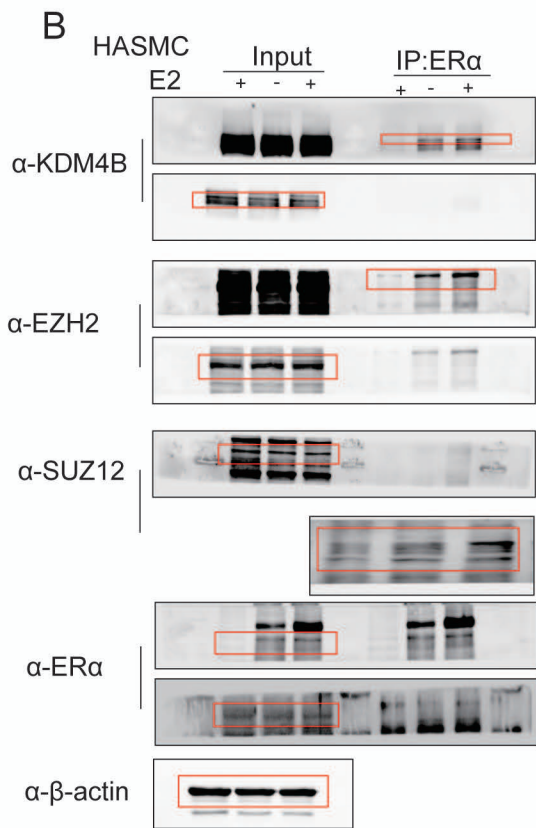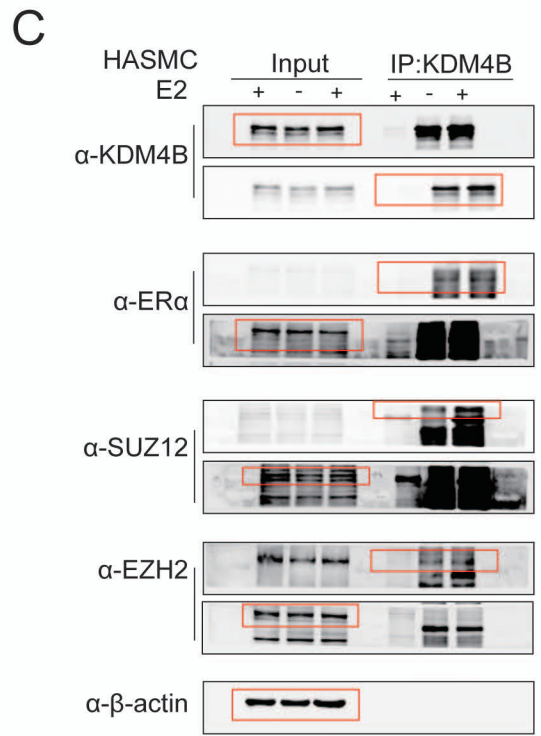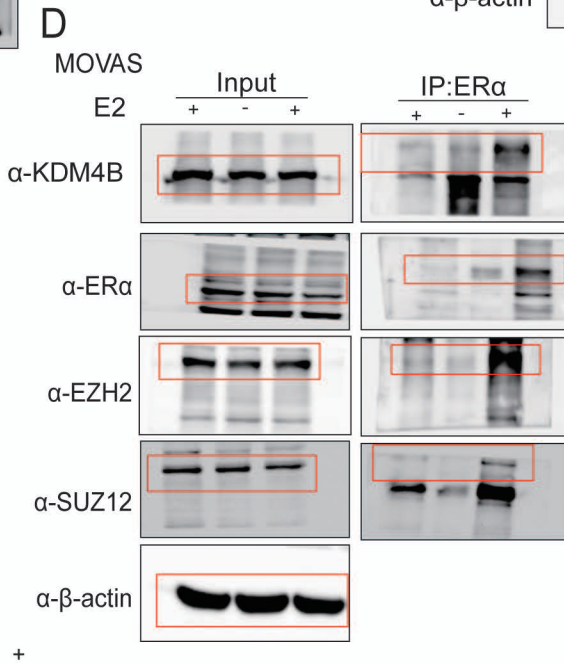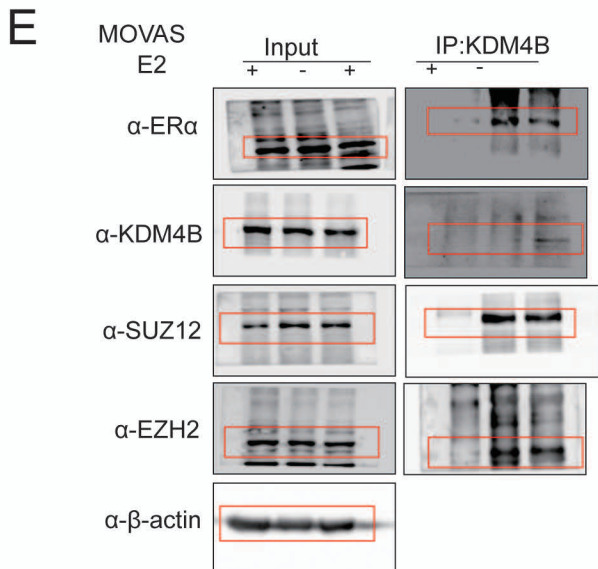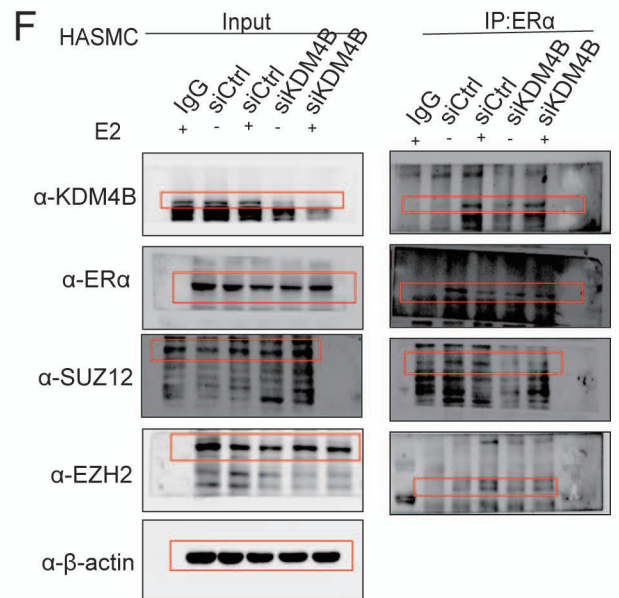

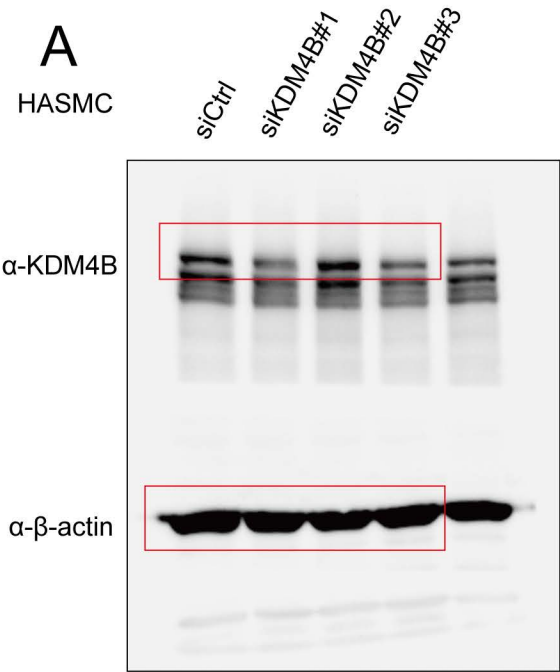

**E**

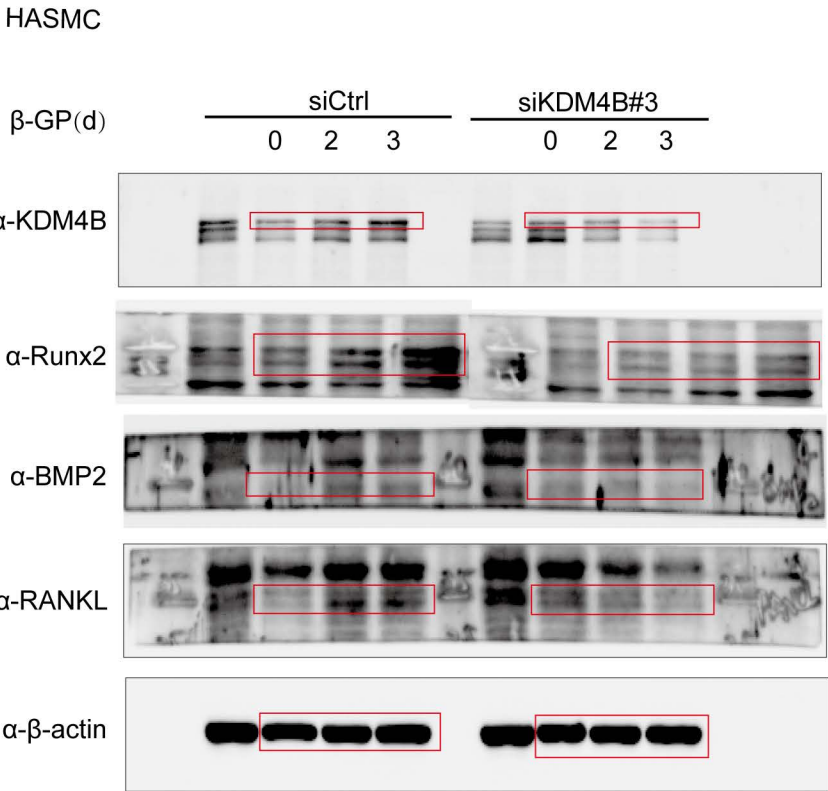

**G**

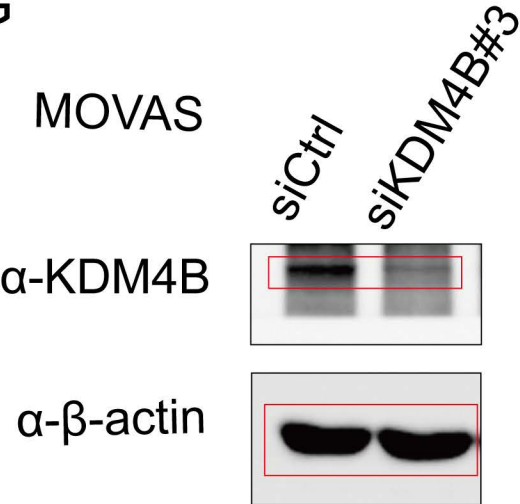

A

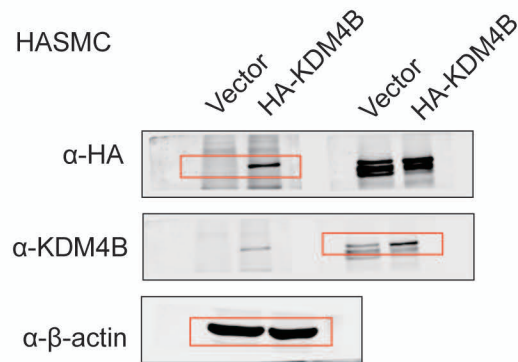

D

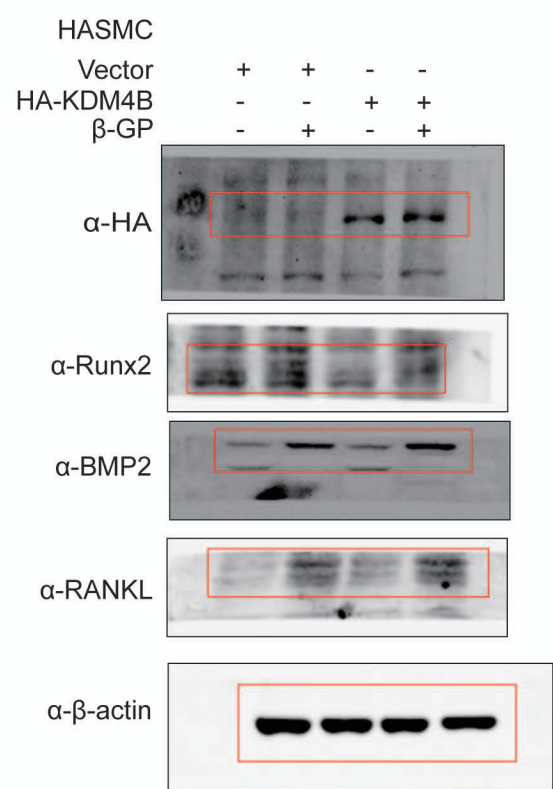

G

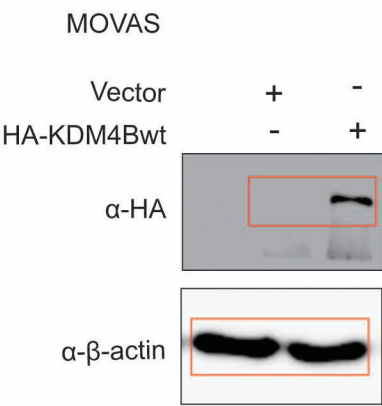

I

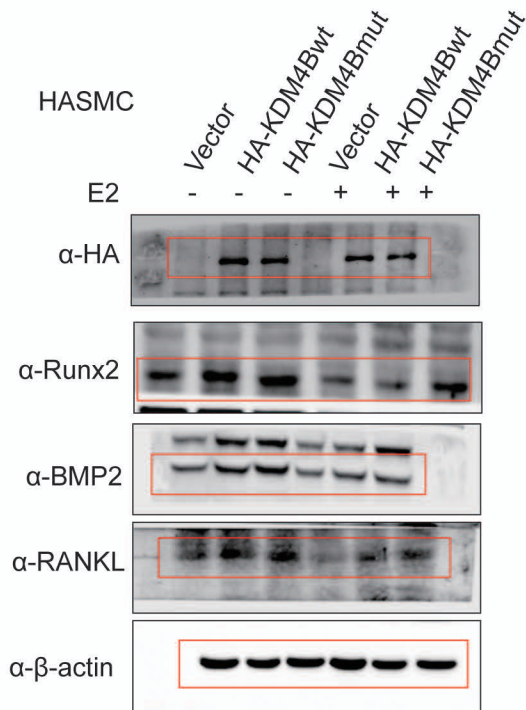

K

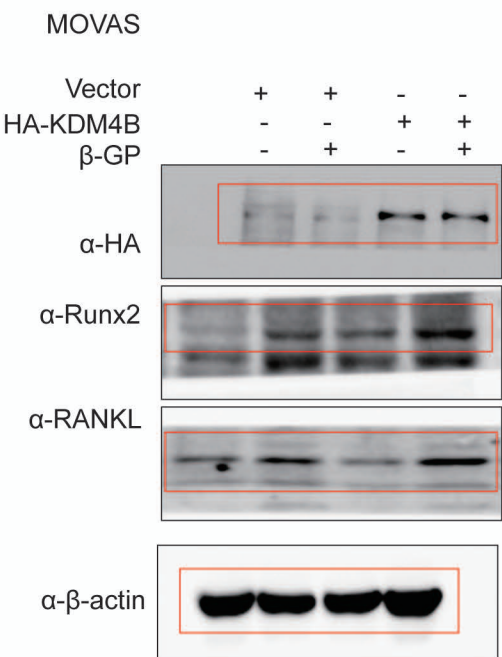

C

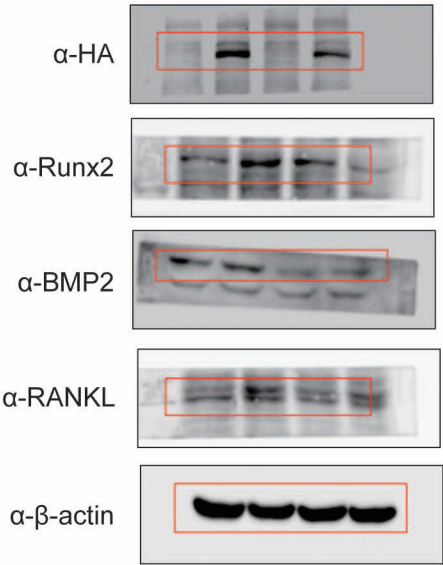

D

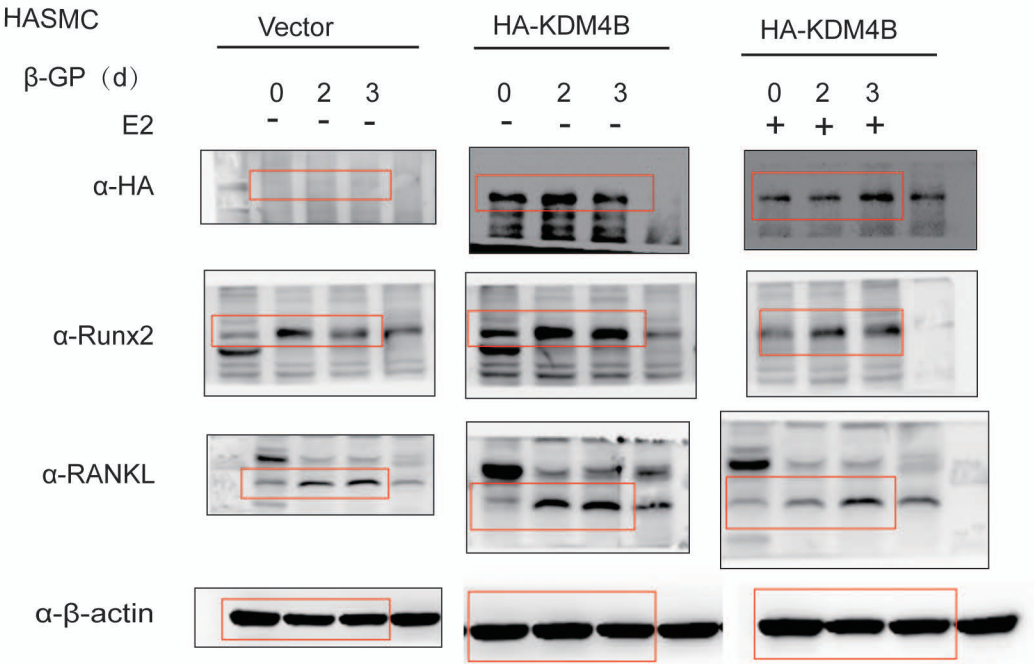

H

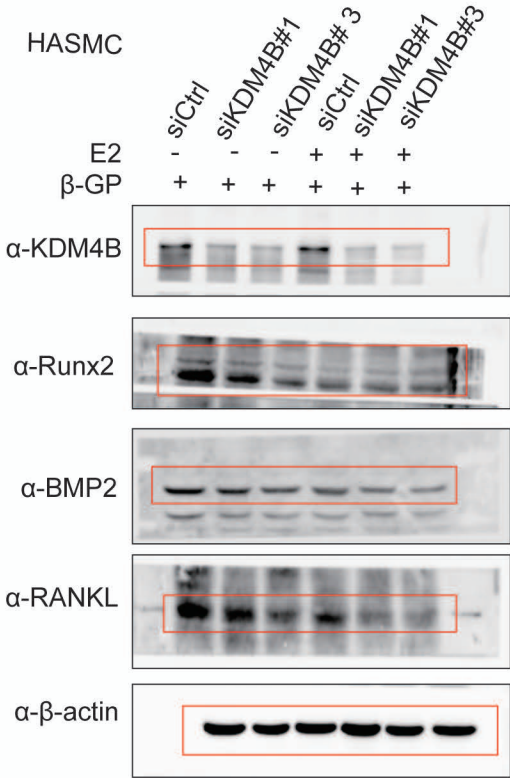

A

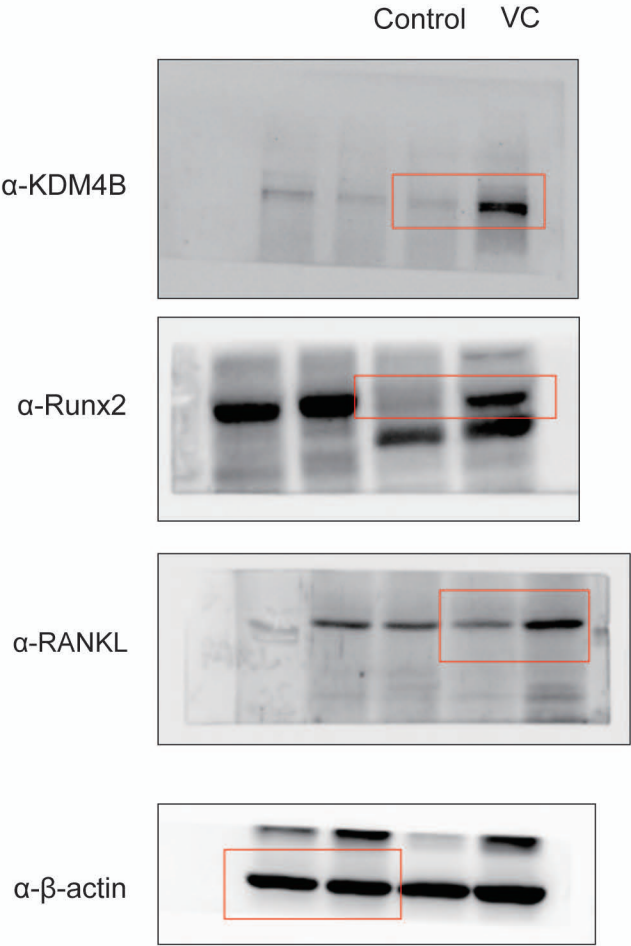

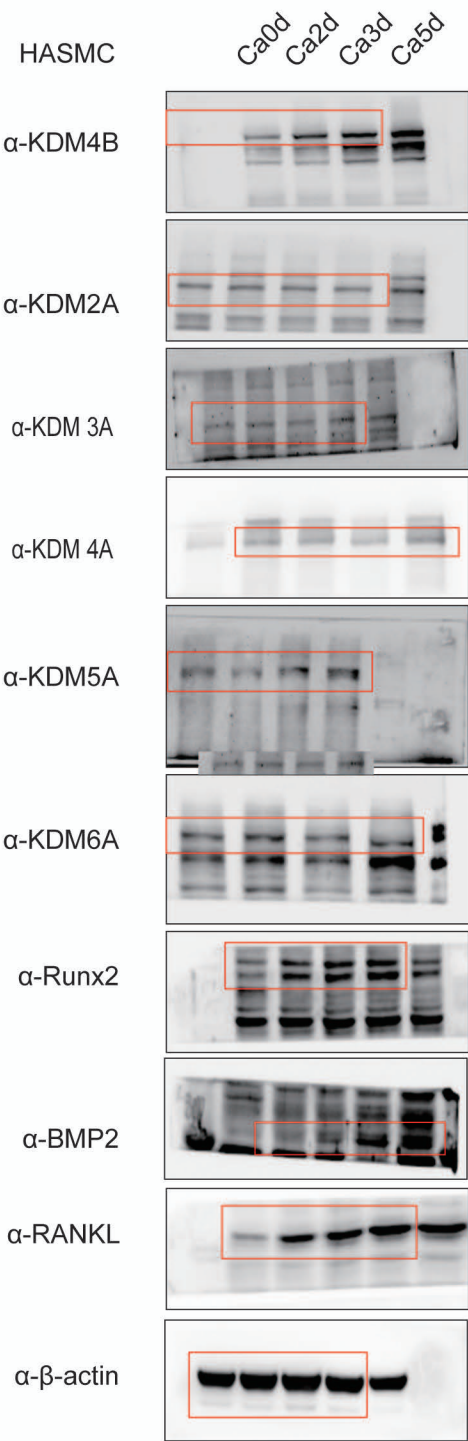

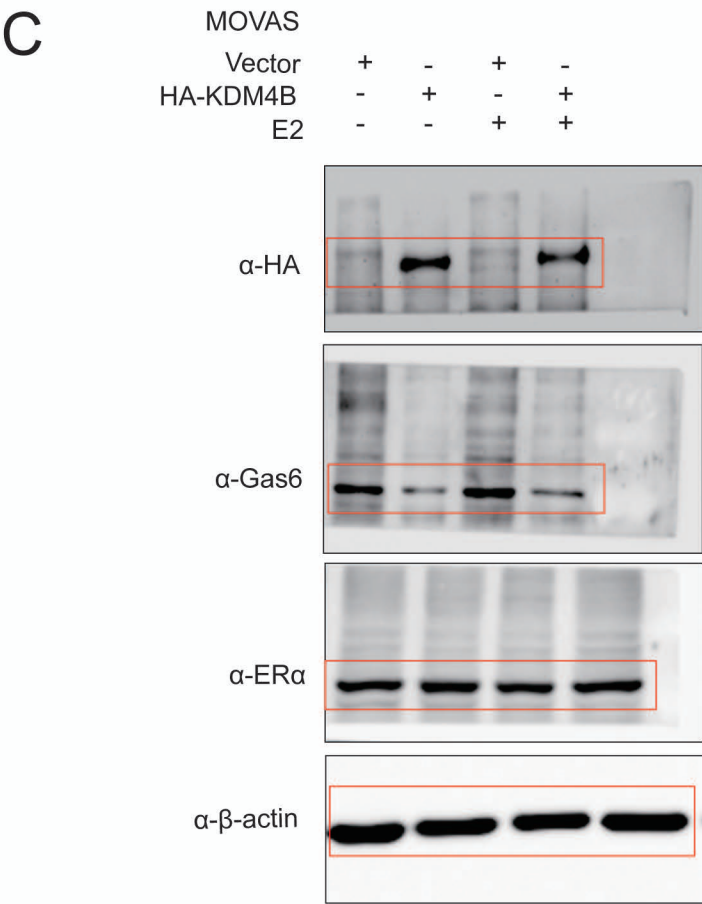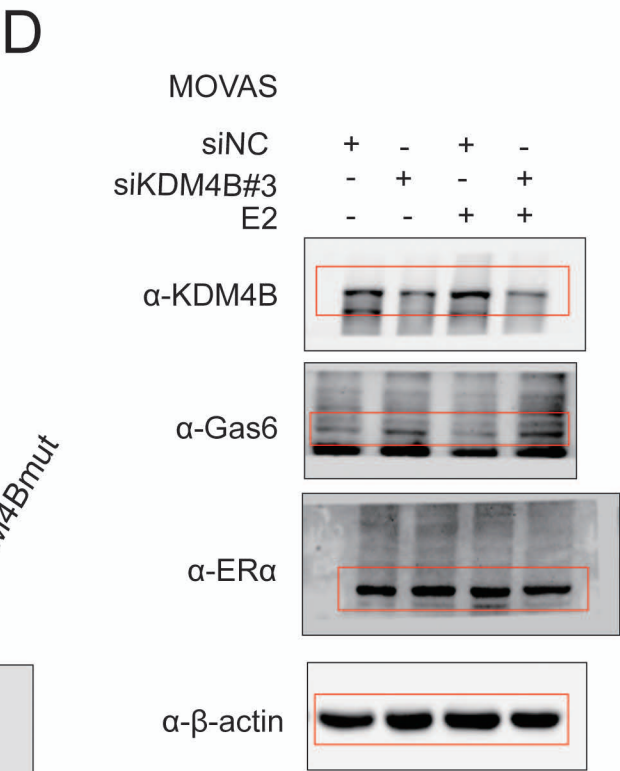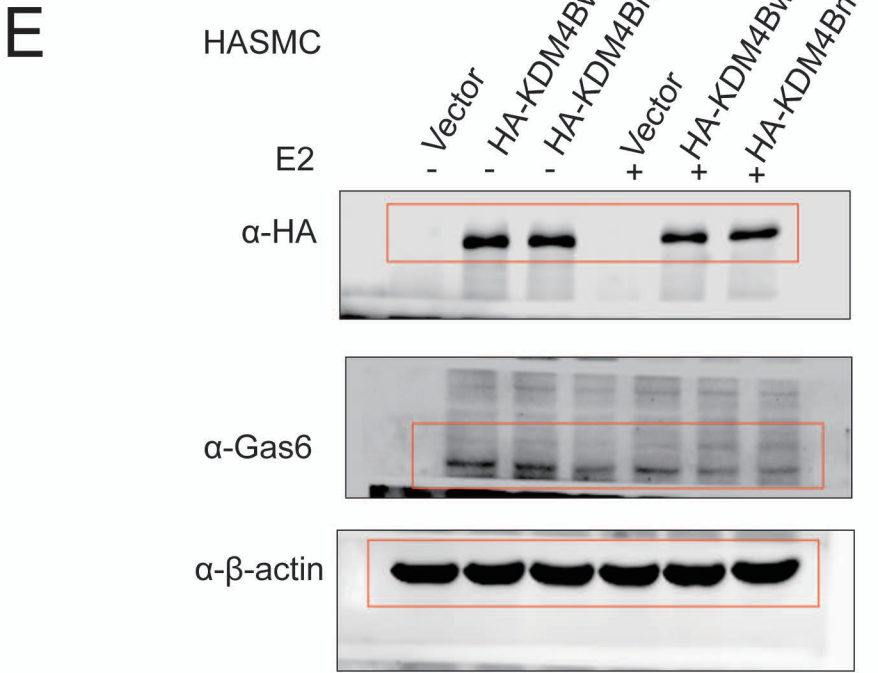

A

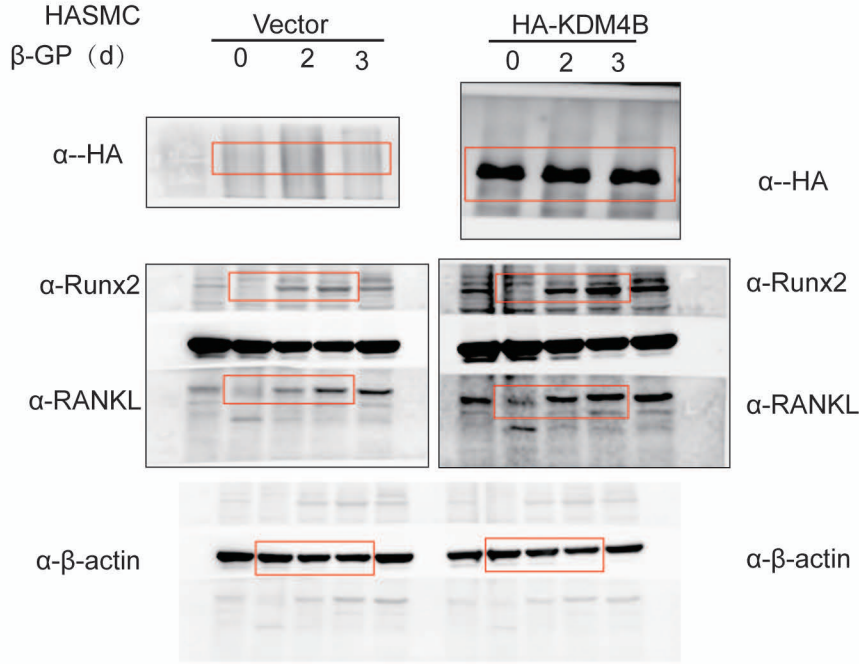

B

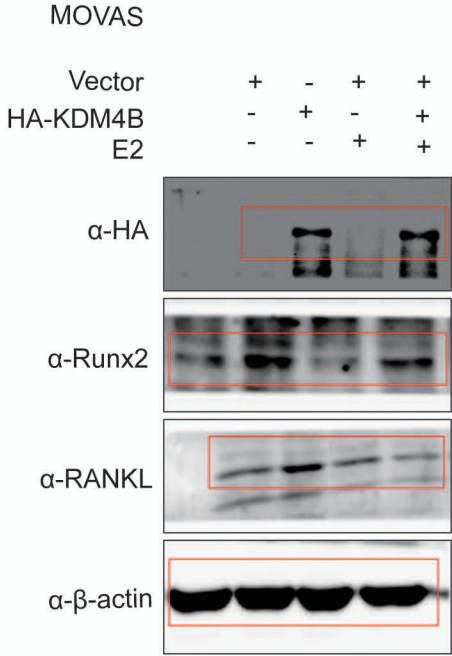

C

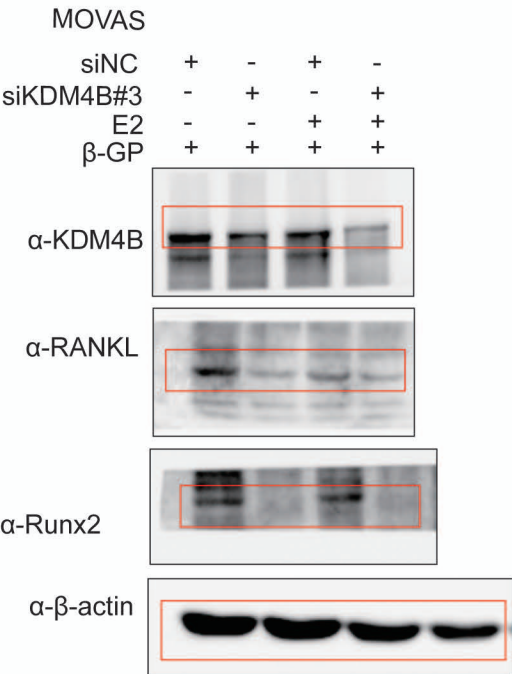

Supplement: Supplementary file 3 — Original Data File [file 41420_2025_2765_MOESM3_ESM.pdf]
